# Meiosis genes in *Daphnia pulex *and the role of parthenogenesis in genome evolution

**DOI:** 10.1186/1471-2148-9-78

**Published:** 2009-04-21

**Authors:** Andrew M Schurko, John M Logsdon, Brian D Eads

**Affiliations:** 1 Roy J Carver Center for Comparative Genomics and Department of Biology, The University of Iowa, Iowa City, Iowa 52242, USA; 2 The Center for Genomics and Bioinformatics and Department of Biology, Indiana University, Bloomington, Indiana 47405, USA

## Abstract

**Background:**

Thousands of parthenogenetic animal species have been described and cytogenetic manifestations of this reproductive mode are well known. However, little is understood about the molecular determinants of parthenogenesis. The *Daphnia pulex *genome must contain the molecular machinery for different reproductive modes: sexual (both male and female meiosis) and parthenogenetic (which is either cyclical or obligate). This feature makes *D. pulex *an ideal model to investigate the genetic basis of parthenogenesis and its consequences for gene and genome evolution. Here we describe the inventory of meiotic genes and their expression patterns during meiotic and parthenogenetic reproduction to help address whether parthenogenesis uses existing meiotic and mitotic machinery, or whether novel processes may be involved.

**Results:**

We report an inventory of 130 homologs representing over 40 genes encoding proteins with diverse roles in meiotic processes in the genome of *D. pulex*. Many genes involved in cell cycle regulation and sister chromatid cohesion are characterized by expansions in copy number. In contrast, most genes involved in DNA replication and homologous recombination are present as single copies. Notably, *RECQ2 *(which suppresses homologous recombination) is present in multiple copies while *DMC1 *is the only gene in our inventory that is absent in the *Daphnia *genome. Expression patterns for 44 gene copies were similar during meiosis *versus *parthenogenesis, although several genes displayed marked differences in expression level in germline and somatic tissues.

**Conclusion:**

We propose that expansions in meiotic gene families in *D. pulex *may be associated with parthenogenesis. Taking into account our findings, we provide a mechanistic model of parthenogenesis, highlighting steps that must differ from meiosis including sister chromatid cohesion and kinetochore attachment.

## Background

Sexual reproduction (*i.e. *meiosis and syngamy) is the predominant reproductive mode in eukaryotes, yet parthenogenesis (*i.e. *asexual reproduction) is present in all major lineages. Among animals, cyclical parthenogenesis, which alternates bouts of clonal and sexual reproduction, is restricted to monogonont rotifers, digenean trematodes, and several arthropod lineages [1]. Obligate parthenogenesis is much more common but is predicted ultimately to drive lineages to extinction due to the accumulation of deleterious mutations or inability to adapt to environmental changes [2]. The origins of obligate parthenogens are often attributed to the loss of meiosis *via *interspecific hybridization [3,4] or irreversible changes in ploidy [5,6], yet other mechanisms must also exist. Among animals, thousands of parthenogenetic species have been described, and volumes have been written describing the cytogenetic manifestations of many different types of parthenogenesis (*e.g. *[7-9]), but little is understood about molecular determinants of these processes.

The microcrustacean *Daphnia pulex *reproduces by cyclical parthenogenesis. Direct-developing eggs (also called subitaneous or summer eggs) are produced parthenogenetically and immediately develop in the female's brood chamber prior to hatching. During the sexual cycle, haploid resting eggs (also called ephippial, diapause, or winter eggs) are produced by meiosis and require fertilization and a period of extended dormancy for development to complete. Because sex determination in *Daphnia *is environmentally induced [10,11], males are genetic clones of their mothers. In addition, genetic and phenotypic evidence has revealed *D. pulex *lineages that reproduce by obligate parthenogenesis. These obligate parthenogenetic lineages produce direct-developing eggs by parthenogenesis, which is indistinguishable from parthenogenesis in cyclical lineages. However, obligate parthenogens have lost the requirement for meiosis and fertilization to produce viable resting eggs [12]; unfertilized resting eggs undergo a period of diapause and develop parthenogenetically to eventually hatch and produce a juvenile. Importantly, the resting egg parthenogenesis exhibited by these obligate asexual lineages is cytologically distinct from direct-developing egg parthenogenesis in both obligate and cyclical parthenogenetic lineages and from meiosis. Hence, although the terms "cyclical parthenogenesis" and "obligate parthenogenesis" may also refer to breeding systems, herein we use these terms to distinguish parthenogenetic oogenesis that takes place during direct-developing (in cyclical and obligate asexuals) and resting egg development (in obligate asexuals only), respectively. Therefore, the *D. pulex *genome must contain the molecular machinery to accommodate various types of reproductive modes: meiosis (male and female) and parthenogenetic oogenesis in both cyclical and obligate parthenogenetic lineages. This feature makes *D. pulex *an ideal model to investigate the genetic basis of parthenogenesis, and its consequences for gene and genome evolution.

Resting egg parthenogenesis in *Daphnia *is cytologically distinct compared to direct-developing egg parthenogenesis (*e.g. *with respect to chromosome morphology and egg size [13]). However, while obligate parthenogenesis apparently involves initial meiotic pairing (but without homologous recombination) followed by a mitotic or mitotic-like division ([13,14]; Tsuchiya and Zolan, pers. comm), neither obligate nor cyclical parthenogenesis seems to be strictly mitotic since a polar body is extruded during cell division, indicative of meiosis [15]. In both cases, heterozygosity is maintained, except in rare instances of loss of heterozygosity presumably caused by mitotic crossing over [16]. Obligate parthenogenesis in *Daphnia *is limited to the *D. pulex *complex (*D. pulex*, *D. pulicaria *and *D. middendorffiana*, *D. tenebrosa*) [17] and to the *D. carinata *complex (*D. thomsoni, D. cephalata*) [17], and at least in some cases, the trait is passed by male offspring of obligate asexuals into sexual backgrounds, implying a sex-limited meiosis suppressor [12]. In *D. pulex*, obligate asexuality has been migrating from northeastern to central North America, and most clonal lineages are estimated to be no more than 12,000 – 120,000 yr [18,19]. Recent association mapping of obligate asexuality in *Daphnia *has found markers on four different chromosomes exhibiting significant association with parthenogenetic production of resting eggs in obligate asexuals [19]. This suggests that obligate asexuality and (by implication) the mechanistic transition from meiosis to parthenogenesis could be influenced by at least four epistatically interacting loci.

Specifically, we are interested in genes that encode components essential for meiosis in *D. pulex*. A cyclically parthenogenetic *D. pulex *lineage possesses genes required for both meiosis and parthenogenesis. To ultimately establish whether modifications to the meiotic machinery are associated with parthenogenesis, we must first determine which meiotic genes are present and expressed in cyclically parthenogenetic lineages. Then, we can compare the inventory and expression patterns of these same genes in obligate parthenogens. If obligate parthenogens have truly abandoned canonical meiosis altogether, genes required specifically for meiosis should be under reduced selective constraint and become non-functional over time. However, certain meiotic processes, perhaps in a modified form, may still be required for parthenogenesis and, thus, genes required for such processes may still be intact and expressed. Differences in the inventory, evolutionary rates and expression of meiotic genes in cyclical and obligate parthenogens may provide insight into the importance of meiotic genes for the evolution of parthenogenesis.

**Figure 1:**
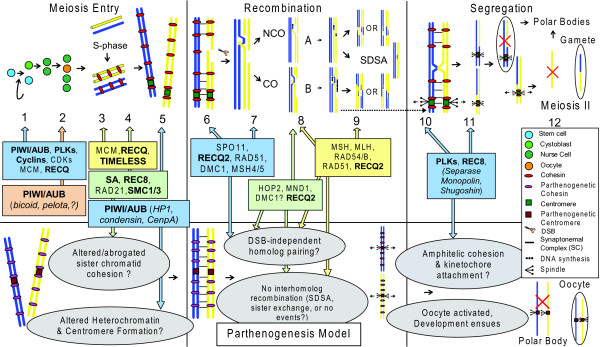
**Meiotic genes annotated in the *D. pulex *genome (shown in boxes) and a schematic of a possible model for parthenogenesis**. Arrows indicate their roles in meiosis, and potentially in parthenogenesis. Proteins in bold are encoded by multiple gene copies in *D. pulex *(some non-annotated genes are italicized; see text for details). A timeline (top) for three stages of meiosis (meiosis entry, recombination, and segregation) is indicated at the top. Meiosis I events from germline stem cell (GSC) division (1) through DSB formation, synapsis and recombination (2–9), kinetochore attachment (10) and anaphase (11) occur during canonical meiosis I. Meiosis II follows (12), with the loss of centromeric cohesion and segregation of sister chromatids resulting in the final haploid gamete. In our model for parthenogenesis (bottom), sister chromatid cohesion somehow differs from meiosis while altered heterochromatin and centromere formation may be important for homolog pairing and segregation. Reciprocal recombination is suppressed and syntelic rather than amphitelic kinetochore attachment is also posited (grey ovals). Our model for parthenogenesis predicts the expulsion of a single diploid polar body after a mitotic cell division accompanies production of the diploid "gamete" which gives rise to the next generation. See text for a more detailed description of the stages of meiosis and explanation of our model for parthenogenesis.

During a typical animal meiosis (Fig. 1), a germline stem-cell (GSC) divides asymmetrically producing a daughter GSC and either a cystoblast (females) or gonialblast (males) [9]. During both meiosis and parthenogenesis in *Daphnia *females, incomplete mitoses create a 4-cell cystoblast which matures into an oocyte cluster of three nurse cells and the presumptive oocyte [15]. Only later in vitellogenesis can parthenogenetically-produced oocytes be distinguished visually from meiotically-produced oocytes [15]. As the oocyte cluster matures, pre-meiotic S-phase DNA replication occurs in the oocyte, followed by heterochromatin and centromere specification and, in most animals, appearance of the synaptonemal complex (SC) [20]. In most organisms studied, cohesin complexes are recruited during S-phase to promote cohesion between sister chromatids [21]. Several mechanisms have been reported to initiate chiasmata formation and recombination between homologous chromosomes, including double-strand break (DSB) formation and DSB-independent pathways [22]. As recombination progresses, syntelic attachment of sister kinetochores (*i.e. *both attached to the same spindle pole) generates monopolar tension towards the spindle poles, leading to segregation of homologous chromosome pairs at anaphase and cytokinesis resulting in two diploid cells [23]. In the second meiotic division, amphitelic attachment of kinetochores (*i.e*. associated with microtubules from opposite spindle poles) and the complete removal of cohesin allow sister chromatids to segregate to opposite poles [23]. As a result, one haploid cell is formed; it becomes the ovum while two polar bodies are produced and eventually degenerate.

While parthenogenesis in *Daphnia *shares some features with meiosis (*e.g. *oocyte cluster formation, extrusion of polar bodies), there are important differences. First, during parthenogenesis sister chromatids segregate in a mitosis-like fashion, suggesting that sister chromatid cohesion must be different. This could be a result of parthenogenesis-specific cohesin complexes or altered timing of cohesin removal. Second, parthenogenetic kinetochore orientation should be amphitelic (bi-oriented, as in mitosis and meiosis II), again to allow pairs of sister chromatids to segregate towards opposite poles. Lastly, recombination likely differs compared to meiosis because heterozygosity is maintained during parthenogenetic reproduction and chiasmata are not observed [15,16]. These changes likely involve a modification of recombination bias away from reciprocal and homologous exchange to between sisters or to no recombination at all [16].

The major stages of meiosis and the genes which are the targets of our inventory in *D. pulex *are indicated in Fig. 1. The genes were chosen with a focus on female meiosis and their potential role(s) in parthenogenesis. In this study, we report an inventory of genes in the genome of a cyclically parthenogenetic strain of *D. pulex *(strain TCO) that encode proteins with roles throughout meiosis. This represents an initial step in identifying and characterizing the genes that are central to reproduction in *D. pulex*. We have divided these meiotic genes intro two broad categories. First, we investigate "meiosis-related genes": these are genes that encode proteins involved in meiosis but whose functions and expression are not specific to meiosis. These include genes encoding Argonaute proteins (PIWI and AGO subfamilies), cell cycle regulation proteins (cyclins, cyclin dependent kinases (CDKs) and polo kinases) and several proteins involved in DNA replication, cohesion and meiotic recombination (minichromosome maintenance (MCM), TIMELESS (TIM) and RecQ proteins). Second, we investigate several meiosis-specific genes in our inventory: these are genes for which homologs in most model organisms function are expressed only during meiosis and mutants containing null alleles are defective only in meiosis. These genes include *SPO11, MND1, HOP2, DMC1, REC8, MSH4*, and *MSH5*, which encode proteins that together generally affect the initiation and progression of meiotic recombination and sister chromatid cohesion. We also examine gene families that are closely involved in the above processes: these include structural maintenance of chromosome (*SMC*) and stromal antigen (*SA*) gene families, *RAD54 *and *RAD54B *paralogs, and eukaryotic homologs of bacterial *mutL *and *mutS *genes. Database homology searches and rigorous phylogenetic analyses are employed to identify orthologs and distinguish paralogs. For 42 gene copies, we use RT-PCR to compare expression levels in ovaries of females undergoing meiosis or obligate (resting egg) parthenogenesis, in males (*i.e. *undergoing meiosis) and in female somatic tissue. We interpret our results from these experiments in light of a model of the genetic underpinnings of parthenogenesis we have developed for *D. pulex*.

The gene inventory and the expression patterns of these genes during meiosis and parthenogenesis will help us address whether parthenogenesis uses existing meiotic and mitotic machinery, or whether novel processes may be involved. While thelytokous parthenogenesis may occur via various cytological mechanisms [9], parthenogenesis in *Daphnia *appears to be apomictic and does not involve gametic fusion as would be observed with automictic reproduction [15]. The transition from meiosis to parthenogenesis in *Daphnia *requires at least three modifications: altered spindle attachment of the kinetochore, modified sister chromatid cohesion and abrogation of homologous recombination (cf. [24]). It is not clear whether any one of these changes is necessary or sufficient for the origin of thelytokous parthenogenesis in *D. pulex*, or whether they are pertinent for other types of parthenogenesis such as arrhenotoky. However, these modifications must involve characterized pathways in mitosis and meiosis, for which mutant phenotypes closely resemble the cytogenetic manifestations characteristic of parthenogenotes [20,21,23,25]. Therefore, our inventory includes genes required for these and other meiotic processes.

## Results and discussion

Our primary goal is to determine whether features of the *D. pulex *genome could account for differences between parthenogenesis and meiosis. The presence/absence, copy number and expression pattern of each meiotic gene will influence our model for parthenogenesis. We have annotated several gene families in *D. pulex *with known functions in the meiotic pathways above. Here we describe duplications of genes relating to several relevant processes, including sister chromatid cohesion, centromere orientation, and crossover suppression during homologous recombination. We also describe the maintenance of single gene copies for many proteins involved in homologous recombination, specifically in DSB formation, recombination initiation, strand invasion and Holliday junction resolution.

### *Daphnia* homologs of *Drosophila *meiotic genes

Initially, we searched for genes in *D. pulex *that have known meiotic roles in *Drosophila melanogaster *(the closest relative to *Daphnia *for which there is extensive genetic and functional data for meiosis) and determined whether these genes are expressed during cyclical parthenogenesis and if gross differences in expression levels were evident when comparing direct-developing (parthenogenetic) and resting (meiotic) egg production. Gene expression was empirically determined by agarose gel electrophoresis of RT-PCR products; amplicons were evidence for gene expression while negative results indicated a lack of expression (Additional File 1). Oogenesis during cyclical parthenogenesis in *Daphnia *does not appear to be mitotic (since a polar body is extruded). Therefore, expression of a meiotic gene during direct-developing egg production could suggest that parthenogenesis requires components of the meiotic machinery. Alternatively, absence of expression could provide insight into which processes in meiosis are absent or modified in parthenogenesis.

In *D. pulex*, we determined the presence or absence of 25 homologs of *Drosophila *meiotic genes from Flybase [26] (Table 1) based on BLAST search results because initially we were interested in whether parthenogenetic reproduction is associated with lack of expression of meiosis-associated genes. RT-PCR from 12 distinct genotypes of pooled whole females revealed that 22 out of 25 of these genes are expressed during *D. pulex *oogenesis (only for *CHK2, MNS1/MUS301 *and *NEBBISH *was expression not detected) (Table 1). Cyclically parthenogenetic females producing either direct-developing or resting eggs demonstrated indistinguishable expression patterns (Table 1). Therefore, gross discrepancies in expression levels of these genes during parthenogenetic and meiotic reproduction in cyclical parthenogens are unlikely to be responsible for the mechanistic differences between these reproductive modes.

**Table 1:** Homologs of select *Drosophila melanogaster *meiosis-associated genes in *D. pulex*.

***D. melanogaster *protein**	**Function**	***Daphnia *Gene Model**	**NR-blast E-value**	**cDNA**
Abnormal × segregation	Female meiosis chromosome segregation	JGI_V11_233883	5 × 10^-112^	Yes

Anaphase Promoting Complex	Ubiquitin ligase	NCBI_GNO_1048014	0	Yes

ATM/Rad3/Mei-41	Kinase, DNA repair and recombination	JGI_V11_299627	0	Yes

Boule	RNA binding; male meiosis	JGI_V11_299633	9 × 10^-15^	Yes

Chk2	Ser/Thr kinase	NCBI_GNO_348374	1 × 10^-15^	No

Courtless	Ubiquitin ligase; male meiosis	JGI_V11_196074	4 × 10^-86^	Yes

Effete	Ubiquitin ligase; meiosis	JGI_V11_69870	5 × 10^-51^	Yes

ERCC4	Excision repair nuclease	JGI_V11_331812	0	Yes

Fumble	Pantothenate kinase; meiosis	JGI_V11_299558	1 × 10^-135^	Yes

Grapes	Checkpoint kinase 1; female chromosome segregation	JGI_V11_43327	1 × 10^-148^	Yes

Kinesin-like protein at 3A	Chromosome-associated kinesin; male meiosis/mitosis	JGI_V11_232248	0	Yes

Lesswright	Ubiquitin conjugation; female meiosis chromosome segregation	JGI_V11_230818	1 × 10^-85^	Yes

Mei-P26	Ubiquitin ligase; meiosis	JGI_V11_299653	5 × 10^-63^	Yes

Meics	Spindle component, transcription factor; meiosis	NCBI_GNO_178964	3 × 10^-64^	Yes

Minispindles	Microtubule binding	JGI_V11_299544	0	Yes

Missing oocyte	Protein binding; meiosis	JGI_V11_301937	0	Yes

Mutagen-sensitive 81	Crossover junction endonuclease	JGI_V11_304957	6 × 10^-91^	Yes

Mutagen-sensitive 301/Mns1	DNA polymerase theta	JGI_V11_320958	3 × 10^-179^	No

Nebbish	Kinesin	JGI_V11_47335	2 × 10^-82^	No

Non-claret disjunctional	Kinesin-like; spindle assembly, chromosome segregation	JGI_V11_299957	5 × 10^-95^	Yes

Out at first	Female meiosis chromosome segregation	JGI_V11_47164	1 × 10^-61^	Yes

Pavarotti	Kinesin; female meiosis chromosome segregation	JGI_V11_305721	0	Yes

Separase	Cohesin proteolysis	JGI_V11_243106	5 × 10^-36^	Yes

String	Tyrosine kinase, meiosis	JGI_V11_44847	6 × 10^-48^	Yes

Transforming acidic coiled coil	Microtubule binding; meiosis, mitosis	NCBI_GNO_562034	5 × 10^-32^	Yes

Genes were detected by BLAST protein homology searches using annotations and literature available at FlyBase [26]. Evidence for gene expression (indicated by "Yes" or "No") was determined using cDNA from pooled genotypes of cyclical parthenogens producing either resting eggs (*via *meiosis) or direct-developing eggs (*via *parthenogenesis). Expression patterns between meiosis and parthenogenesis were indistinguishable.

In the course of cataloging these genes in *D. pulex*, we found gene copy number expansions for many additional meiotic genes and gene families. We have divided these genes into two categories: I) Meiosis-related genes and II) Meiosis-specific genes (see Table 2). These genes were subject to rigorous phylogenetic analyses and, in many cases, expression studies which are discussed in the following sections.

**Table 2:** Summary of the presence, absence and number of copies of annotated meiotic genes in *Daphnia pulex*.

**I. Meiosis-related Genes**			**II. Meiosis-specific Genes**		
**Gene**	Present (+)/Absent (-)	Copy No.	**Gene**	Present (+)/Absent (-)	Copy No.

**A) Argonaute Proteins**			**A) Cohesin Protein Families**		

*AUB/PIWI*	+	6	*SMC1*	+	2

*AGO1*	+	1	*SMC2*	+	1

*AGO2*	+	1	*SMC3*	+	2

*AGO3*	+	6	*SMC4*	+	1

**B) Cell Cycle Proteins**			*SMC5*	+	1

*Cyclin A*	+	1	*SMC6*	+	2

*Cyclin B*	+	5	*RAD21*	+	1

*Cyclin B3*	+	1	*REC8*	+	3

*Cyclin D*	+	2	*Stromal Antigen*	+	5

*Cyclin E*	+	1	**B) Interhomolog Recombination Proteins**		

*CDK1 (CDC2)*	+	1	*SPO11*	+	1

*CDK2*	+	1	*MND1*	+	1

*CDK4*	+	1	*HOP2*	+	1

*CDK10*	+	1	*RAD54*	+	1

*PLK1*	+	3	*RAD54B*	+	1

*PLK2/3*	+	1	*RAD51*	+	1

*PLK4*	+	1	*DMC1*	-	0

**C) Replication Factors**			*RAD51B*	+	1

*MCM2*	+	1	*RAD51C*	+	1

*MCM3*	+	1	*RAD51D*	+	1

*MCM4*	+	1	*XRCC2*	+	1

*MCM5*	+	1	*XRCC3*	-	0

*MCM6*	+	1	**C) Mismatch Repair Proteins**		

*MCM7*	+	1	*MSH2*	+	1

*MCM8*	+	1	*MSH3*	+	1

*MCM9*	+	1	*MSH4*	+	1

*TIMELESS* *(TIM-1)*	+	9	*MSH5*	+	1

*TIMEOUT* *(TIM-2)*	+	2	*MSH6*	+	1

*RECQ1*	+	1	*MLH1*	+	1

*RECQ2*	+	7	*MLH2*	+	1

*RECQ3*	-	0	*MLH3*	+	1

*RECQ4*	+	1	*PMS1*	+	1

*RECQ5*	+	1			

**Figure 2:**
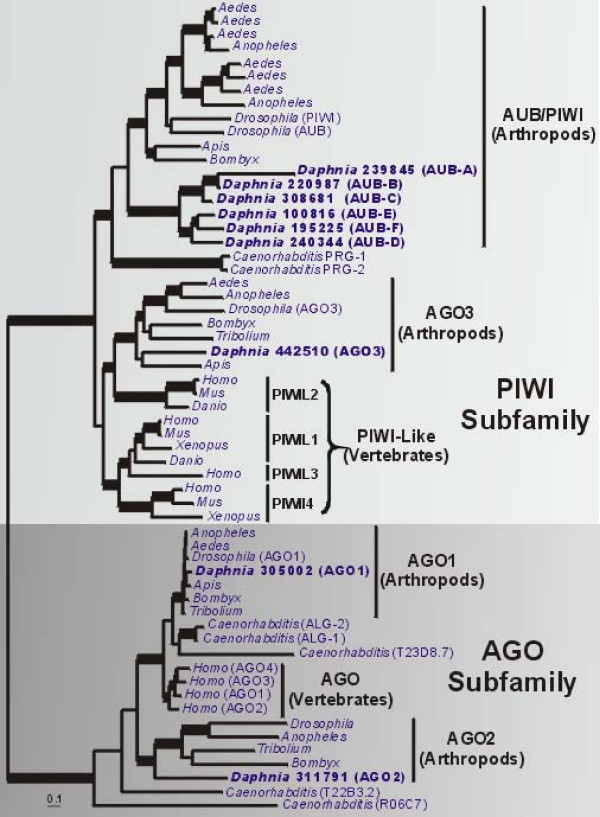
**Bayesian phylogenetic analysis of Argonaute family proteins in the PIWI and AGO subfamilies**. Phylogeny is based on an alignment of 714 amino acids and the tree shown is a consensus of 951 best trees. Parameter means: α = 2.13, pI = 0.01 and lnL = -54179.80. Thickened branches refer to posterior probabilities from 0.95 to 1.0. Protein identifiers for *D. pulex *sequences (in bold) are in Additional File 2.

**Figure 3:**
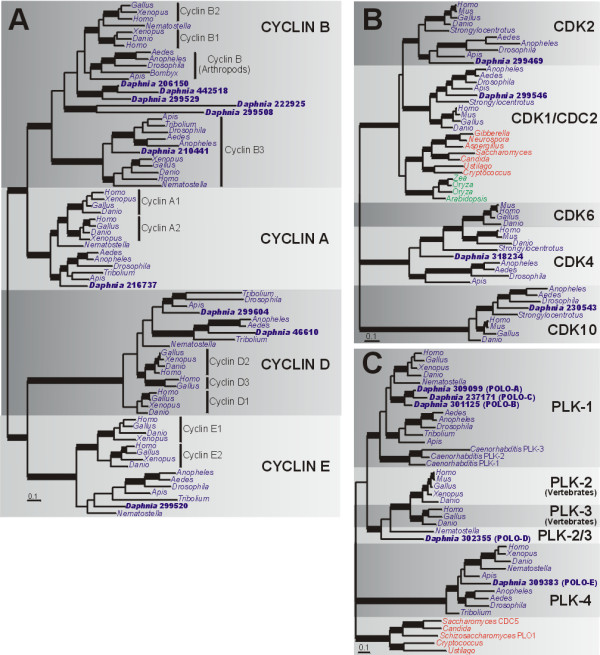
**Bayesian phylogenetic analyses of cell cycle proteins**. (a) Phylogeny of cyclin A, B, D and E proteins. Tree shown (consensus of 951 best trees) is based on an alignment of 189 amino acids. Parameter means: α = 1.78, pI = 0.05 and lnL = -16804.13. (b) Phylogeny of CDK proteins. Tree shown (consensus of 951 best trees) is based on an alignment of 285 amino acids. Parameter means: α = 1.22, pI = 0.12 and lnL = -12586.10. (c) Phylogeny of PLK proteins. Tree shown (consensus of 931 best trees) is based on an alignment of 250 amino acids. Parameter means: α = 1.33, pI = 0.1 and lnL = -10286.85. Blue, red and green names indicate animals, fungi and plants, respectively. Thickened branches refer to posterior probabilities from 0.95 to 1.0. Protein identifiers for *D. pulex *sequences (in bold) are in Additional File 2.

**Figure 4:**
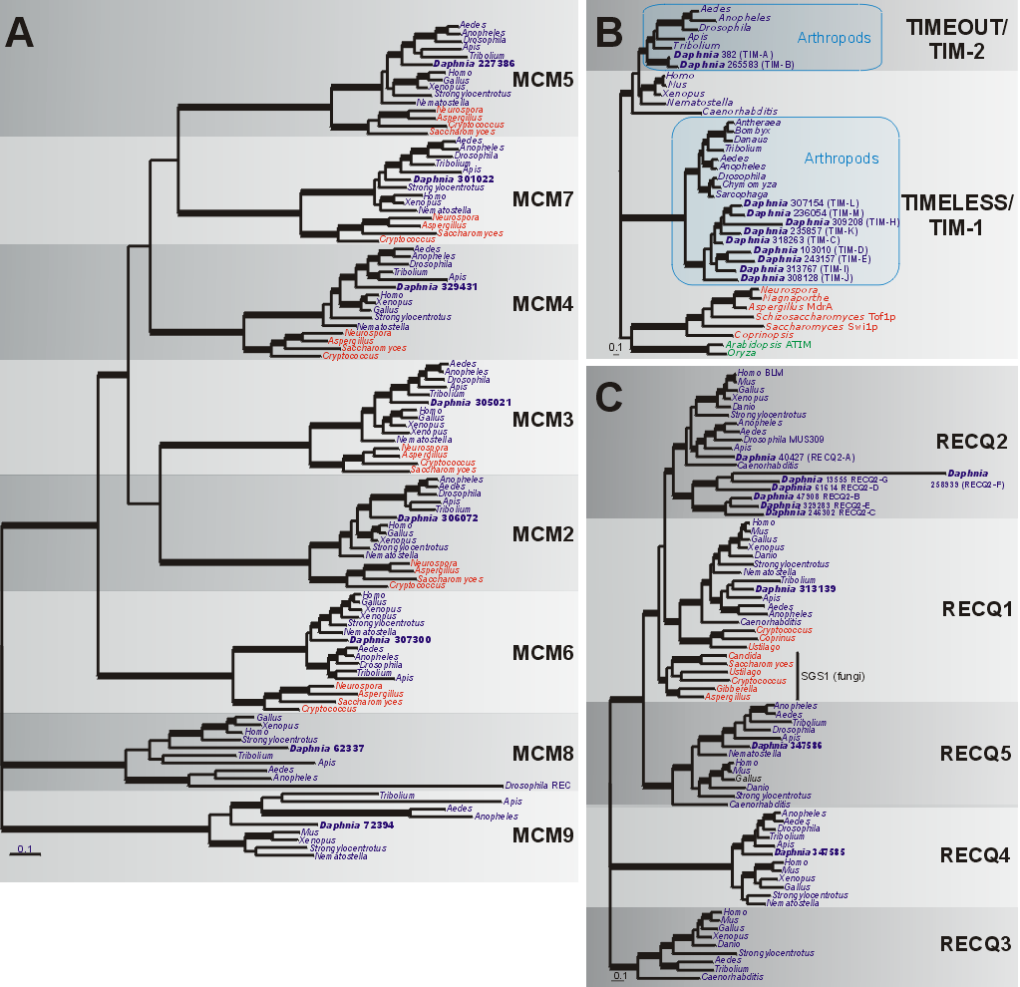
**Bayesian phylogenetic analyses of DNA replication regulatory proteins**. (a) Phylogeny of MCM proteins. Tree shown (consensus of 931 best trees) is based on a phylogeny of 490 amino acids. Parameter means: α = 1.25, pI = 0.03 and lnL = -48902.97. (b) Phylogeny of TIMELESS (TIM-1) and TIMEOUT (TIM-2) proteins. Tree shown (consensus of 951 best trees) is based on an alignment of 491 amino acids. Parameter means: α = 3.77, pI = 0.003 and lnL = -28413.38. (c) Phylogeny of RECQ protein family. Tree shown (consensus of 951 best trees) is based on an alignment of 370 amino acids. Parameter means: α = 1.25, pI = 0.045 and lnL = -34130.50. Blue, red and green taxa names indicate animals, fungi and plants, respectively. Thickened branches refer to posterior probabilities from 0.95 to 1.0. Protein identifiers for *D. pulex *sequences (in bold) are in Additional File 2.

**Figure 5:**
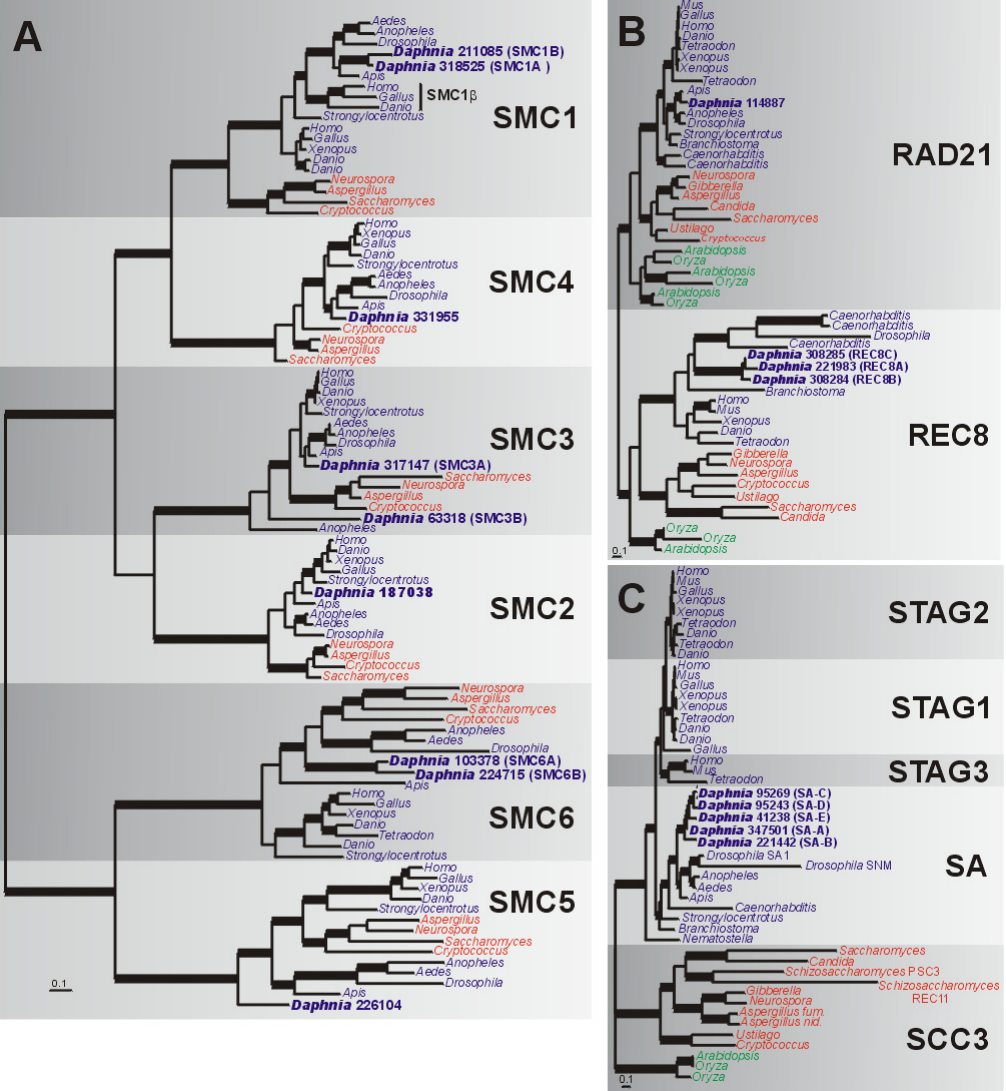
**Bayesian phylogenetic analyses of cohesin complex proteins**. (a) Phylogeny of SMC family proteins based on an alignment of 255 amino acids. Parameter means: α = 1.75, pI = 0.036 and lnL = -23686.88. (b) Phylogeny of RAD21 and REC8 proteins based on an alignment of 141 amino acids. Parameter means: α = 1.86, pI = 0.033 and lnL = -10212.86. (c) Phylogeny of stromal antigen (SA) proteins based on an alignment of 651 amino acids. Parameter means: a = 3.06, pI – 0.01 and lnL = -34655.82. For all analyses, the tree shown is a consensus of 951 best trees. Blue, red and green names indicate animals, fungi and plants, respectively. Thickened branches refer to posterior probabilities from 0.95 to 1.0. Protein identifiers for *D. pulex *sequences (in bold) are in Additional File 2.

**Figure 6:**
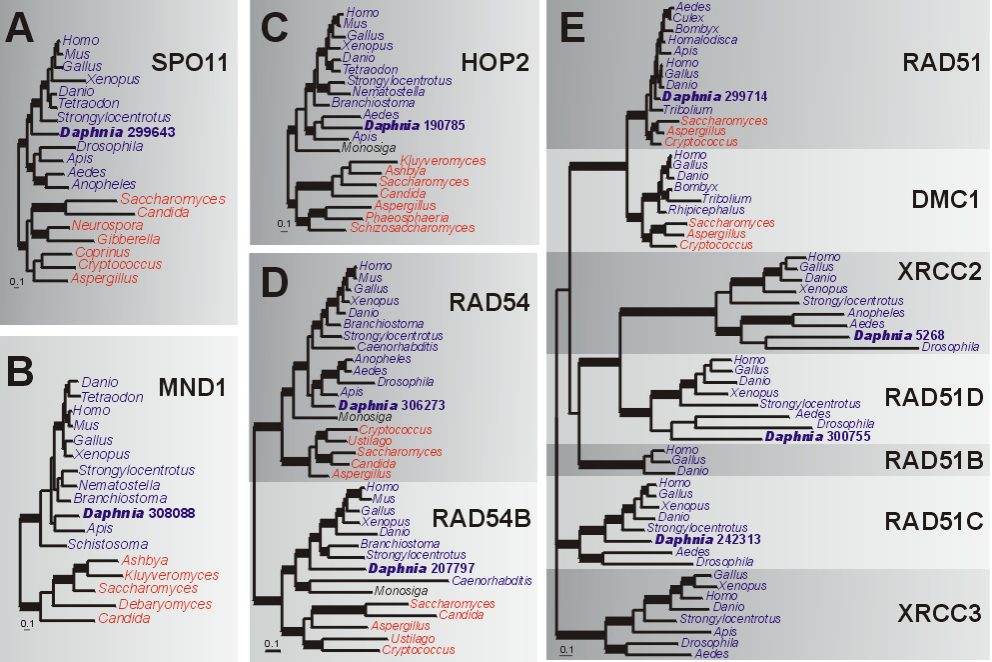
**Bayesian phylogenetic analyses of interhomolog recombination proteins**. (a) SPO11 phylogeny based on an alignment of 284 amino acids. Parameter means: α = 2.16, pI = 0.019 and lnL = -9924.87. (b) MND1 phylogeny based on alignment of 205 amino acids. Parameter means: α = 1.87, pI = 0.024 and lnL = -5532.70. (c) HOP2 phylogeny based on an alignment of 192 amino acids. Parameter means: α = 3.23, pI = 0.022 and lnL = -6807.46. (d) RAD54/RAD54B phylogeny based on an alignment of 485 amino acids. Parameter means: α = 1.27, pI = 0.14 and lnL = -17500.27. (e) Phylogeny of RAD51-like family proteins based on an alignment 232 amino acids. Parameter means: α = 2.21, pI = 0.017 and lnL = -18082.74. For all analyses, the tree shown is a consensus of 951 best trees. Blue, red and black names indicate animals, fungi and choanoflagellates, respectively. Thickened branches refer to posterior probabilities from 0.95 to 1.0. Protein identifiers for *D. pulex *sequences (in bold) are in Additional File 2.

**Figure 7:**
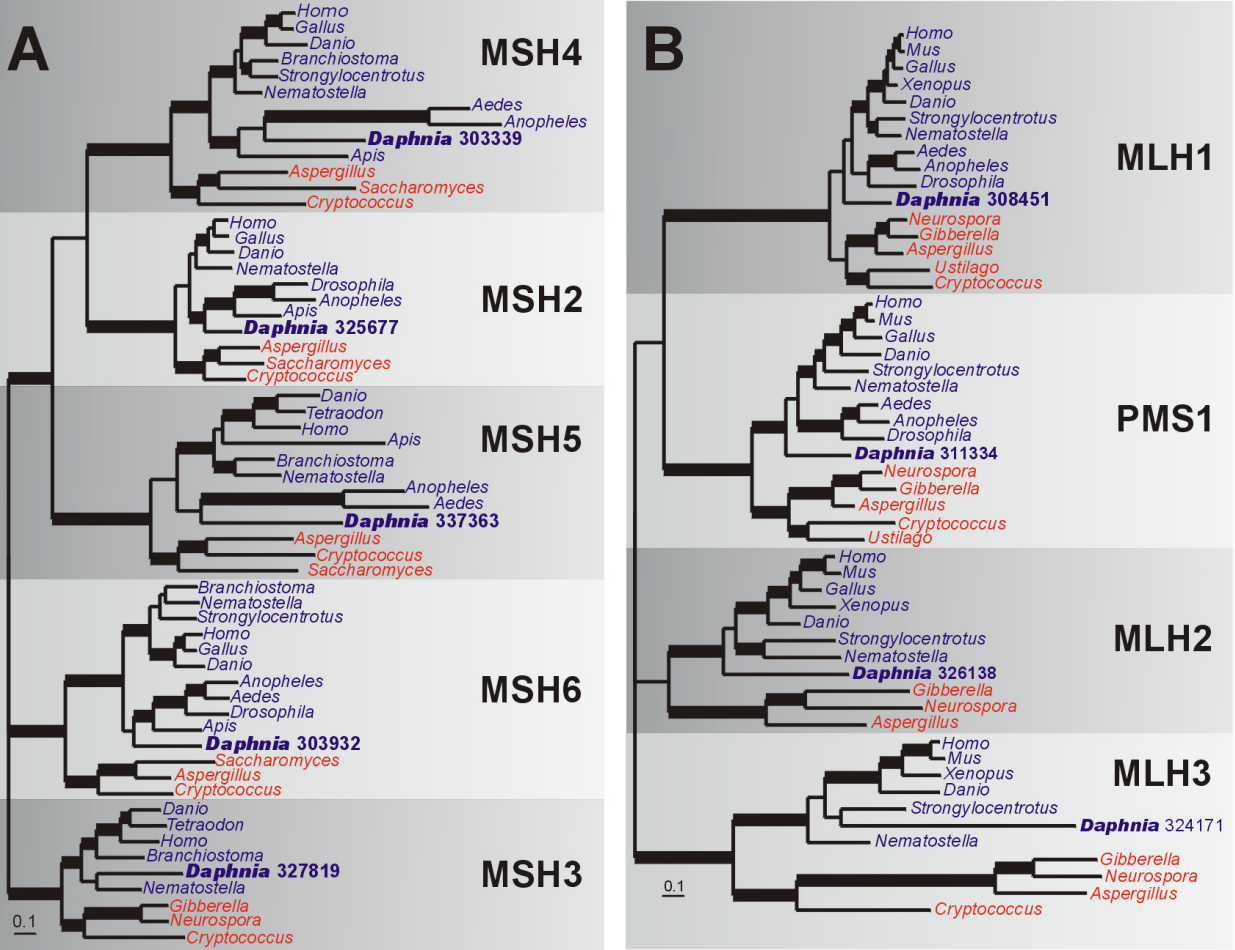
**Bayesian phylogenetic analyses of mismatch repair proteins**. (a) Phylogeny of MutS homologs (MSH2-6) based on an alignment of 327 amino acids. Parameter means: α = 1.79, pI = 0.04 and lnL = -28040.35. (b) Phylogeny of MutL homologs (MLH1, 2, 3 and PMS1) from an alignment of 330 amino acids. Parameter means: α = 2.2, pI = 0.031 and lnL = -24034.03. For both (a) and (b), the tree shown is a consensus of 951 best trees. Blue and red taxa names indicate animals and fungi, respectively. Thickened branches refer to posterior probabilities from 0.95 to 1.0. Protein identifiers for *D. pulex *sequences (in bold) are in Additional File 2.

See Figures 2 to 7 for phylogenetic positions of all gene copies. Protein IDs, genomic coordinates and expression data for each gene copy is available in Additional File 2. Partial gene sequences and putative pseudogenes are not included below, but are discussed in the text and also provided in Additional File 2.

#### I) Meiosis-related genes

##### A) Argonaute Proteins (PIWI and AGO subfamilies)

The Argonaute protein family is comprised of the PIWI and AGO subfamilies. These proteins bind distinct subsets of small (24–31 nt) repeat-associated RNAs (also called rasiRNAs or piRNAs) [27] and constitute core elements of the RNA-induced silencing complex (RISC) (reviewed in [28]). A central function of Piwi subfamily proteins is transposon control in the germ line mediated *via *binding piRNAs, which has been shown to be important for normal meiosis and germ cell development, but other roles in chromatin formation and (indirectly) kinetochore specification are likely. The production of pachytene piRNAs in mouse, which are depleted of transposon sequences, also indicates roles for Piwi subfamily proteins beyond transposon control [28]. In *Drosophila*, the PIWI subfamily protein aubergine (AUB) has demonstrated roles in piRNA binding and DNA damage signaling, and this family is implicated in a range of other processes as well [29]. Independent experiments using microarrays to monitor gene expression during resting egg production in sexual and obligate asexual *D. pulex *(Eads and J. Andrews, unpub.) also revealed some copies of this family to be differentially expressed, prompting us to conduct a more thorough phylogenetic analysis of these proteins.

For *D. pulex*, the Argonaute protein family phylogeny distinguishes seven PIWI and two AGO subfamily proteins, each of which is encoded by individual genes (Fig. 2). Among the PIWI subfamily proteins, six (AUB-A to AUB-F) form a clade within the larger AUB/PIWI clade, indicating multiple gene duplications have occurred in the *Daphnia *lineage. Duplications are also present in other arthropod lineages and *Caenorhabditis*. The seventh protein (442510) is present among arthropod AGO3 homologs. *D. pulex *also has single *AGO1 *and *AGO2 *homologs (protein IDs 305002 and 311791, respectively) closely related to arthropod orthologs of these genes. The three *D. pulex *proteins not included in the initial analysis (442513, 130069 and 317739) are truncated copies which are difficult to align and likely represent pseudogenes; a subsequent phylogenetic analysis revealed strong support for 442513 and 130069 within the arthropod AGO1 clade, and a long branch for 317739 within the *Daphnia *AUB/PIWI clade (tree not shown).

##### B) Cell cycle proteins: Cyclins, CDKs, Polo kinases

The cyclins and their regulatory counterparts, cyclin-dependent kinases (CDKs), have important roles in the mitotic cell cycle and in meiotic cell division (for reviews, see [30,31]). There are four cyclin families (cyclins A, B, D and E) for which mutants exhibit meiotic defects in mammals [20], and we searched the *D. pulex *genome for homologs of these proteins. Cyclins A and B are involved in M-phase progression and regulation. For example, in *Drosophila*, cyclin A stability controls progression of cystoblast divisions and oocyte cell fate [32]. Cyclins D and E are part of the G1 complex. Cyclin D has important roles in cell proliferation and regulation of the cell cycle, transcription, histone acetylation and chromatin remodeling [33]. In mammals, data from null mutants reveal important roles for cyclin D in follicle cell maturation and spermatogenesis [20]. Cyclin E is involved in G1 to S phase progression by its regulatory association with CDK2 and also has a CDK-independent role in DNA replication by regulating activity of the replicative MCM helicase [34].

There are a total of ten homologs encoding cyclins A, B, D, and E in *D. pulex *(Table 2). In the phylogenetic analysis of animal cyclin proteins (Fig. 3A), cyclins A, B, D, and E each form strongly supported clades and earlier duplications gave rise to the cyclin A/B and D/E lineages. The single *D. pulex *cyclin A (216737) is related to arthropod sequences within the cyclin A clade, and its gene model is supported by both cDNA and tiling path evidence (data available at wFleabase; see Additional File 2). The cyclin B family is larger, containing one cyclin B3 homolog (210441) and five additional gene copies encoding cyclin B (Fig. 3A). Among these five copies, proteins 222925 and 299508 form a strongly supported clade, but their long branch lengths indicate rapid evolution, suggesting a possible long branch attraction artifact. When 299508 is removed from the analysis, 222925 appears as a long branch within the vertebrate cyclin B2 clade (tree not shown). However, when 222925 is omitted, 229508 does not show strong affinity for any cyclin classes (tree not shown). This is consistent with the difficulty we had in aligning 299508, and this protein is the only cyclin B copy without expression data (Additional File 2), and for which we were unable to validate expression using primers derived from the gene model (for primer data see Additional File 3). Thus, while the gene encoding 299508 possibly represents a pseudogene, the divergent cyclin B homolog 222925 presents an interesting case for further study.

For cyclin D, arthropod homologs are distinguished from clades formed by vertebrate cyclins D1, D2 and D3 (Fig. 3A). *D. pulex *has two copies encoding cyclin D proteins (299604 and 46610) which are related to other arthropod homologs. Most other arthropods in the analysis (except *Tribolium*) have only one cyclin D copy (Fig. 3A). While vertebrates typically have two distinct cyclin E homologs, E1 and E2, invertebrates usually contain only one. The single cyclin E copy in *D. pulex *(299520) is related to invertebrate homologs, although its position among arthropods is not strongly supported.

Cyclin-dependent kinases (CDKs), the regulatory binding partners of cyclins, have roles in cell-cycle progression through meiosis and are synthesized and degraded in a cell cycle-dependent manner (reviewed in [31]). CDK1 (called CDC2 or CDC28 in fungi) regulates G2-M phase progression and interacts with A-and B-type cyclins. Defects in meiosis I spindle assembly have been demonstrated for *CDK1 *mutants [31]. CDK2 mainly interacts with A and E-type cyclins (and cyclin B1 and some D-type cyclins in mammals) to regulate G1 to S progression. Chromosome pairing is defective and meiosis is incomplete when *CDK2 *is absent in mammalian cells [35]. CDK4 and CDK6 regulate progression through G1 to S phase and interact with D-type cyclins [36]. CDK10 regulates the G2-M phase and inhibits transactivation of the Ets2 transcription factor which regulates *CDK1 *expression [37]. We characterized homologs encoding four CDK family members in *D. pulex*: CDK1 and CDK2 (the cell cycle-related kinases), CDK4/6 and CDK10.

In *D. pulex*, there are single gene copies of each *CDK *family member (Table 2). In the phylogeny (Fig. 3B), each CDK protein forms a strongly supported clade. *D. pulex *proteins 299546 and 299469 cluster with arthropod CDK1 and CDK2 proteins, respectively. The tree topology suggests a vertebrate-specific *CDK4/CDK6 *gene duplication, and the relationship of the *D. pulex CDK4 *homolog (318234) with either vertebrate or arthropod homologs is unresolved. Invertebrate CDK10 protein sequences, including *D. pulex *CDK10 (230543), have long branch lengths compared to vertebrates (Fig. 3B). The *CDK *gene family is not markedly expanded in *D. pulex *(in contrast to cyclin genes with which they interact for their roles in cell cycle regulation).

Polo kinases (PLKs) have well-characterized roles in regulating mitotic cell-cycle progression and spindle attachment to kinetochores during meiosis [23]. Polo-like kinase 1 (*PLK1*) is present in many eukaryotes and is the best studied of the group. In yeast, the PLK1 homolog CDC5 promotes spindle co-orientation, chiasmata resolution and meiosis I exit (reviewed in [31,38]). In addition, phosphorylation of the cohesin subunit SA2 by PLK1 is critical for cohesin removal during prophase [23]. While evidence suggests that PLK2, PLK3 and PLK4 are also involved in cell cycle progression, their functions are less well-understood.

We found eleven *PLK *homologs in *D. pulex *(Additional File 2); however, six homologs (*POLO-F *to *POLO-K*) were removed from the phylogenetic analysis because they were either too short and/or were difficult to align (see below). The phylogeny (Fig. 3C) shows that *D. pulex *has at least three gene copies encoding PLK1 and single copies each for PLK2/3 and PLK4 (Table 2). For *PLK1*, multiple independent gene duplications are evident in *D. pulex *and *C. elegans *lineages. *D. pulex *proteins POLO-A, B and C form a clade but their relationship relative to other animals is unresolved. *POLO-A *and *POLO-C *are separated by almost 1 Mb on scaffold 9. For *PLK2 *and *PLK3*, vertebrate gene copies evolved *via *an ancient gene duplication (Fig. 3C); the *D. pulex *(POLO-D) and *Nematostella *proteins (designated PLK2/3) are basal to the vertebrate PLK2/3 clade and *PLK2/PLK3 *orthologs were lost in the other insects examined. *D. pulex POLO-E *is present among other arthropod sequences in the PLK4 clade.

Six putative PLK homologs initially removed from the alignment (POLO-F to POLO-K; Additional File 2) were included in a separate PLK1, PLK2 and PLK3 phylogenetic analysis (not shown). POLO-F to POLO-K were present within the PLK1 clade with strong support, however these proteins apparently do not represent complete and intact genes (which tend to encode ~550–600 aa proteins in animals). However, these copies evidently contain stop codons (*POLO-G*) or re-arrangements (*POLO-F *and *POLO-H*). Therefore, these partial *PLK *copies appear to be pseudogenes, or remnants of partial gene duplications, based on: i) lack of expression evidence, ii) apparent gene chimerism or rearrangements, iii) truncated length, and/or iv) premature stop codons.

##### C) Replication factors: MCM, Tim, RecQ

Sister chromatid cohesion is normally established early in DNA replication (reviewed by [39]) and because cohesion is likely more dynamic and highly regulated than most models would indicate [40], a wide variety of replication factors may influence cohesin loading. The establishment and maintenance of sister chromatid cohesion plays a critical role in our model for parthenogenesis in *D. pulex *(Fig. 1). In this section, we focus on proteins involved in regulating DNA replication and establishing and maintaining sister chromatid cohesion. Specifically, we search for homologs within the minichromosome maintenance (MCM) family of replication factors, the TIMELESS family (TIMELESS/TIM-1 and TIMEOUT/TIM-2) and the RECQ family of DNA helicases.

Within the MCM family of DNA helicases (reviewed in [41]), six members (MCM2-7, the replicative MCMs) are structurally related and function together as a hexameric helicase in DNA replication. Additionally, the MCM2-7 complex has been implicated in DNA damage response, chromatin structure and transcription [42]. In vertebrates, MCM8 (which does not associate with MCM2-7) may function in elongation during DNA replication [43], but in *Drosophila*, MCM8 (called REC) facilitates crossovers during meiosis [44]. A function for MCM9 has not been determined.

Our phylogenetic analysis reveals that the *D. pulex *genome contains single copies for each of the eight *MCM *genes (*MCM2 *to *MCM9*, Fig. 4A and Table 2); *D. pulex *sequences usually cluster with respective arthropod sequences with strong support. The replicative MCMs (MCM2-7) share a common ancestor, consistent with the hypothesis that they arose early in eukaryotic evolution [44]. The relationships of MCM8 and MCM9 (which have apparently been lost in fungi) are unresolved. Within the MCM8 clade, the *Drosophila *REC branch is much longer compared to those for other animals. This is consistent with the novel meiotic recombination role for REC in *Drosophila*, compared to the ancestral DNA helicase function in vertebrates [43]. MCM9 has been reported to be vertebrate-specific [45]; however, we found *MCM9 *orthologs in arthropods (except *Drosophila*) including a single copy in *D. pulex*. For MCM9, arthropod branch lengths are very long compared to those for vertebrates. This could suggest that the invertebrate lineage of MCM9-like proteins has evolved a new function (similar to REC in *Drosophila*), or simply that MCM9 proteins are more widespread in eukaryotes than originally suggested.

TIMELESS (TIM-1) is a circadian rhythm protein in insects [46,47], while the function of the TIM-related protein TIMEOUT (TIM-2) has not been clearly defined [47]. In mammals, TIM and its binding partner, TIPIN, maintain replication fork integrity during both challenged (*e.g. *across damaged sites) and normal DNA synthesis [47]. In *C. elegans*, TIM physically interacts with SMC1 of the cohesin complex and directly regulates the loading of cohesin during S-phase [48]. Genetic screens have also implicated *TIM *and *TIPIN *orthologs in meiotic chromosome segregation, although their exact roles are unclear [49].

Our phylogeny (Fig. 4B) clearly distinguishes insect TIMELESS/TIM-1 and TIMEOUT/TIM-2 lineages and shows non-insect animal TIM proteins are homologs of insect TIMEOUT/TIM-2, consistent with previous results [47]. The relationships of the fungal and plant clades of TIM-like sequences relative to the animal clades are unclear. It is possible that genes encoding TIM-1 and TIM-2 represent an animal-specific duplication in the TIMELESS family. In *D. pulex*, we found 13 genes (*TIM-A *to *TIM-M*, Additional File 2) with homology to TIM-related proteins; among these there are two (*TIM-A *and *TIM-B*) *TIMEOUT/TIM-2 *and nine (*TIM-C *to *TIM-M*) *TIMELESS/TIM-1 *homologs (Table 2); *TIM-F *and *TIM-G *were omitted because they encode very short sequences and were difficult to align. The nine TIMELESS protein homologs (TIM-C to TIM-M) form a strongly-supported clade among the arthropod copies, indicating that multiple *TIMELESS/TIM-1 *gene duplications have occurred in the *Daphnia *lineage. This is the first evidence that the *TIMELESS/TIM-1 *gene family is present in arthropods other than insects (blue shading in Fig. 4B). The *D. pulex *TIMEOUT/TIM-2 protein homologs (TIM-A and TIM-B) are closely related to each other and to insect TIMEOUT homologs; however, TIM-B (310 aa) is much shorter than TIM-A (1097 aa).

The *RECQ *gene family of DNA helicases has conserved roles in DNA replication and recombination (reviewed in [50,51]). Unicellular eukaryotes tend to have one *RECQ *homolog (*e.g. SGS1 *in *S. cerevisiae*) while multicellular organisms possess several copies [51]. This gene family receives particular attention for its association with human diseases characterized by premature aging, cancer and genomic instability. These syndromes are caused by mutations to *RECQ2 *(Bloom's syndrome), *RECQ3 *(Werner's syndrome) and *RECQ4 *(Rothmund-Thomson syndrome) [52,53]. *RECQ2 *is the best-studied member of the *RECQ *gene family. RECQ2 proteins (BLM in humans, MUS309 in *Drosophila*, SGS1 in yeast) suppress crossing-over during meiotic prophase I and resolve double Holliday junctions (dHJs) without reciprocal recombination [51,54,55]. The timing and localization of RECQ2 with other proteins involved in meiotic recombination resolution (*i.e. *RAD51, DMC1, RPA, MLH1) are consistent with its role in crossover and recombination regulation [56]. In *D. pulex*, we searched for homologs of these five *RECQ *gene family members.

The phylogeny of animal and fungal RECQ protein homologs (Fig. 4C) shows that *D. pulex *has single gene copies of *RECQ1*, *RECQ4 *and *RECQ5 *and several *RECQ2 *gene homologs (RECQ2-A to RECQ2-G) (Table 2 and Additional File 2). *RECQ3 *is absent in *D. pulex *and may also be absent in other insects. Within the RECQ1 lineage, there are two fungal clades, one corresponding to SGS1 (the fungal RECQ2 homolog) and another which a basidiomycete-specific clade of RECQ1 homologs. The unresolved relationships among RecQ paralogs may require additional sampling of other lineages to determine the order of divergence.

For RECQ2, *D. pulex *RECQ2-A is present within the arthropod RECQ2 clade. The remaining six *D. pulex *proteins (RECQ2-B to RECQ2-G) form a *Daphnia*-specific clade that is sister to other RECQ2 homologs; these proteins are much shorter than RECQ2-A, and many likely represent pseudogenes (see below). RECQ2-A contains DEXDc, Helicase C, RQC and HRDC domains (the last two domains are mutated in Bloom's syndrome patients). This, together with expression data (see below) and its phylogenetic position, indicates that RECQ2-A is likely the functional RECQ2 homolog. The six shorter RECQ2 proteins (RECQ2-B to RECQ2-G) only contain the DEXDc and Helicase C terminal domains, suggestive of roles in RNA metabolism. However, the *RECQ2-B *gene model (NCBI_GNO_1400199) predicts an uncharacteristically large 1.9 kb GC/AG intron near the 5'-end and much of the translation is unalignable and contains multiple stop codons. EST sequences match the region well but continuous ORFs are absent, suggesting *RECQ2-B *is a pseudogene. RECQ2-F (258939) is also unusual: this protein has a very long branch in the phylogeny and BLAST searches suggest strong bacterial homology, although there are apparently numerous introns within the gene. *RECQ2-F *is likely a pseudogene, perhaps the result of a prokaryotic horizontal transfer accompanied by intron gain.

##### D) Expression patterns among meiosis-related gene copies

Among PIWI subfamily members, expression of all *D. pulex *gene copies is well-supported by cDNA and/or tiling path expression data except for protein 130069, which is likely encoded by a pseudogene (Additional File 2). Our RT-PCR expression studies (Additional File 1) demonstrate that *D. pulex AUB-B, AUB-C, AUB-D *and *AGO3 *are expressed in males and in ovaries of cyclical and obligate parthenogens while *AUB-E *and *AUB-F *exhibited expression only in ovaries. *AUB-B, AUB-C *and *AGO3 *were expressed in female soma (Additional File 2), in contrast to the situation in most other eukaryotes, in which PIWI subfamily gene expression is restricted to germ cells [57]. Clear roles for this family of proteins in animal meiosis have not been demonstrated (as they have in plants; see [58]).

Among the cell cycle proteins, tiling path and/or EST evidence showed all cyclin A, B, D and E gene copies are expressed except 299508 (cyclin B) which is consistent with the idea that this copy is a pseudogene (see above). There is also EST and tiling path expression data for all four *CDK *genes in *D. pulex *(Additional File 2). For PLK genes, EST and tiling array data show *POLO-A *and *POLO-B *expression in males and in ovaries during meiosis and parthenogenesis (Additional File 2). However, only *POLO-A *is expressed in somatic tissue and may therefore represent a "mitotic" copy. For *POLO-D *and *POLO-E*, there is EST and tiling expression data and our RT-PCR results show that these genes are expressed in males and in ovaries of obligate and cyclical parthenogens (Additional File 2). Among the more divergent and truncated PLK sequences removed from the phylogenetic analysis (POLO-F To K), there is EST and/or tiling expression evidence for only *POLO-F, POLO-G *and *POLO-H*.

Among the replication factor genes, expression of all *MCM *copies is supported by EST and tiling path data, except *MCM8 *which only has EST data (Additional File 2). Among the *D. pulex TIMELESS/TIM-1 *homologs, there are ESTs and/or tiling array data for *TIM-C, TIM-I, TIM-J *and *TIM-K *while only *TIM-A *has tiling path data for the *TIMEOUT/TIM-2 *homologs. Our RT-PCR experiments showed tissue-specific expression patterns for *TIM-C, TIM-D *and *TIM-E*; *TIM-C *is expressed in female gonads and soma and in males, *TIM-D *only in female gonads and *TIM-E *in males and female gonads but not soma (Additional File 2). Such patterns could be consistent with sub-functionalization. The lack of expression for *TIM-F *and *TIM-G*, together with their truncated protein sequences, suggests that these copies are pseudogenes and perhaps remnant duplications of nearby full-length *TIMELESS/TIM-1 *genes. EST expression evidence for *TIM-L *(also encoding a truncated protein) indicates this partial gene sequence may retain residual function. There is EST and/or tiling expression data for *RECQ1*, *RECQ4 *and *RECQ5 *in *D. pulex*. Dramatic differences in gene expression of *RECQ1*, *RECQ4 *and *RECQ5 *in *D. pulex *were not observed; RT-PCR showed these genes were expressed in parthenogenetic (cyclical and obligate) females, males and female soma. For *RECQ2 *copies, EST and/or tiling array data shows that *RECQ2-A*, *REC2Q-B *and *REC2Q-C *are expressed (Additional File 2). However, RT-PCR shows that *RECQ2-A *is expressed in soma and during obligate parthenogenesis and meiosis, while *RECQ2-B* and *RECQ2-C*are only expressed in female gonads (Additional File 2).

#### II) Meiosis-specific genes

Genes that are meiosis-specific have been experimentally shown to be indispensable for and generally expressed only during meiosis in model organisms [59], but otherwise not to affect organismal viability. Determining the presence and absence of genes that encode proteins functioning only in meiosis will help us to understand the mechanisms of meiosis in *D. pulex*. We have divided the meiosis-specific genes in this study into three broad categories based on their roles in i) sister chromatid cohesion (*REC8*), ii) meiotic interhomolog recombination (*SPO11, MND1, HOP2, DMC1*) and iii) crossover control/resolution (*MSH4, MSH5*). In addition, we search for genes encoding RAD54/RAD54B, stromal antigens and eukaryotic MutL homologs (MLH1, MLH2, MLH3, PMS1), which, while not meiosis-specific, are initially involved in meiotic processes.

##### A) Cohesin gene families: SMCs, RAD21/REC8 and stromal antigens

Cohesin is a multi-protein complex that maintains sister chromatid cohesion until the onset of anaphase in mitosis and meiosis. Cohesin complexes consist of SMC1 and SMC3 (structural maintenance of chromosome proteins), RAD21 (SCC1 or MCD1 in some fungi) or its meiosis-specific paralog REC8, and the stromal antigen protein (SA or STAG in animals, SCC3 or PSC3/REC11 in fungi) (reviewed by [39]). In one well-supported model, RAD21/REC8 binds the globular ATPase ends of SMC1 and SMC3, joining them together in a ring-like structure [60]. The specific roles of SA proteins are less well understood [61,62].

Cohesin is normally loaded onto chromosomes during S-phase [39], although it can also bind to chromosomes independently of DNA replication in response to DSB-induced damage after S-phase [63,64]. Removal of cohesin is generally a two-step process. During vertebrate mitosis, dissociation of cohesin from chromosome arms depends on phosphorylation by the protein kinases PLK1 [65] and Aurora-B [66]. Centromeric cohesin is removed by separase cleavage of RAD21 in a securin-dependent manner which allows anaphase to proceed [31]. During meiosis, RAD21 is largely replaced by its meiosis-specific paralog REC8 [25]; the majority of cohesin along chromosome arms is removed by separase during meiosis I, but centromeric cohesin is protected from cleavage by Shugoshin [67,68]. This protection disappears during meiosis II when separase cleaves centromeric REC8 and cohesin is released, allowing sister chromatids to segregate to opposite poles. For *D. pulex*, we searched for genes encoding SMC1, SMC3, RAD21, REC8 and SA proteins. Sequences for cohesin accessory factors PDS5 [69], separase, securin and Shugoshin are generally poorly conserved in eukaryotes and were not included (although we did identify a putative separase homolog in *D. pulex*; see Table 1).

In eukaryotes, the SMC family of proteins contains six members (SMC1-6) that combine to form heterodimeric complexes. SMC proteins are characterized by two nucleotide binding Walker motifs (A and B) within globular N and C-termini which are separated by a pair of acidic coiled-coil regions joined at the non-helical "hinge" region. Cohesin proteins contain SMC1 and SMC3, while SMC5 and SMC6 (along with several non-SMC components) are part of a DNA repair complex with checkpoint function [70,71]. Condensin complexes contain SMC2 and SMC4, and are involved in chromosome condensation and segregation [72] and in sister kinetochore orientation [23]. In animals and plants, two different condensin complexes (condensin I and II) possess the same core subunits but are distinguished by their regulatory subunits [73].

The phylogeny of animal and fungal SMC homologs reveals that each SMC protein forms a strongly-supported clade (Fig. 5A and Table 2). There is strong support for a duplication that gave rise to the SMC1/4 lineage, but weaker support for the SMC2/3 duplication. SMC5 and SMC6 form a separate group and longer branch lengths compared to other SMCs, suggesting a rapid rate of evolution, which could be related to their unique roles in DNA repair and cell cycle checkpoints. Indeed, SMC5 and SMC6 in *Drosophila *may be under relaxed selection, since they experience higher amino acid substitution rates compared to other SMCs [74].

In *D. pulex*, there are single copies of genes encoding SMC2, SMC4 and SMC5 proteins. SMC2 and SMC4 are closely related to arthropod copies, but SMC5 (226104) is quite divergent and appears as the basal branch among animal SMC5 homologs (Fig. 5A). There are also duplications of genes for SMC1 (SMC1A and SMC1B), SMC3 (SMC3A and SMCB) and SMC6 (SMC6A and SMC6B) in *D. pulex*. The *SMC1 *duplication in *D. pulex *is independent of the vertebrate SMC1 duplication that gave rise to meiosis-specific SMC1β [75]. *SMC1A *and *SMC1B *are on different scaffolds (scaffolds 25 and 20, respectively) while *SMC6A *and *SMC6B *are 165 Kb apart on scaffold 20 (Additional File 2), perhaps the result of an ancient tandem duplication. *SMC3A *and *SMC3B *are distantly related to one another; SMC3B has a longer branch and is present outside of the metazoan/fungal *SMC3 *clade along with a duplicated *SMC3 *from *Anopheles*. Genes for SMC3A and SMC3B differ dramatically in exon composition (21 exons in *SMC3A *and 14 exons in *SMC3B*) but both copies contain a central hinge domain flanked by conserved N- and C-terminal ATPases. These divergent *SMC3 *copies in *D. pulex *and *Anopheles *may represent paralogs that have gained unique cellular function(s); further taxon sampling across all eukaryotes can address this question. We also found nine short protein sequences in *D. pulex *(SMC1C to SMC1K; Additional File 4) ranging from 50 to 306 aa which, based on BLAST search results, represent short domains within SMC1 that have been copied and dispersed throughout the genome. These short coding regions are not generally transcribed, although some loci are imperfect matches for ESTs from the sequenced libraries. Further comparative sequencing and examination of their expression patterns may reveal potential regulatory or functional roles for these small genes.

In the RAD21 and REC8 phylogeny, homologs for each protein form strongly supported clades (Fig. 5B). For *D. pulex*, there is one RAD21 homolog and three genes copies encoding meiosis-specific REC8 (REC8A, REC8B and REC8C; Fig. 5B and Table 2), which are closely related to other arthropod sequences. In animals, *RAD21 *and *REC8 *are typically present as single copies although there are exceptions (*e.g. C. elegans *has multiple copies of both *RAD21 *and *REC8*). For *D. pulex*, *REC8A *is on scaffold 7 while *REC8B *and *REC8C *are found in a head-to-head orientation on scaffold 77 separated by about 12 kb and likely represent a very recent tandem duplication (>98% identical at the DNA level, including introns). *REC8 *copies on scaffolds 7 and 77 are also very similar (about 90% at the DNA and protein level). We cloned and sequenced *REC8 *from cDNA and corrected inaccurate gene models 308284 and 308285 on scaffold 77, and model 221983 on scaffold 7. Our cDNA sequence indicates a protein containing 15 exons comprising 2,016 nucleotides and 671 amino acids. In addition, we found two different regions, one downstream of *REC8A *and one in between *REC8B *and *REC8C*, that each contains distinct sequences found many times within the *D. pulex *genome. Neither region has expression evidence or encodes ORFs, but appear to correspond to repetitive DNAs found at dozens of locations throughout the genome (Additional File 5).

For the *SA *gene family, there have been multiple independent duplications during eukaryotic evolution. In vertebrates, at least two duplications gave rise to *STAG-1*, *STAG-2 *and meiosis-specific *STAG-3 *paralogs [76] and an independent duplication in *Drosophila *resulted in *SA *and meiosis-specific *SNM *paralogs [62]. In fungi, only *S. pombe *shows evidence of an *SCC3 *duplication (giving rise to *PSC3 *and meiosis-specific *REC11 *[61]). In the SA protein phylogeny (Fig. 5C), animal, fungal and plant SA homologs form independent clades and the gene duplications specific to vertebrates (*STAG1-3*), *Drosophila *(*SA/SNM*) and *S. pombe *(*PSC3/REC11*) are evident. In *D. pulex*, there are five SA homologs (Fig. 5C and Table 2) that form a clade closely related to arthropods. Two pairs of the *Daphnia *SA genes (*SA-C/SA-D *and *SA-A/SA-E*) are in a tandem duplication on scaffold 3, while the fifth copy (*SA-B*) is on scaffold 5. This *SA *gene expansion in *Daphnia *is the largest example characterized in eukaryotes; thus, an obvious question is whether one of the copies has a meiosis- or parthenogenesis-specific role (like STAG3 in vertebrates, REC11 in fungi or SNM in *D. melanogaster*).

##### B) Interhomolog recombination genes

Meiotic recombination between homologous chromosomes begins with the creation of double strand breaks (DSBs) to initiate chromosomal synapsis and subsequent interhomolog crossing-over. SPO11, the eukaryotic homolog of an archaeal topoisomerase VI subunit [77], is a trans-esterase that creates these DSBs [22,78]. SPO11 appears to be indispensable for meiosis since homologs have been found in all eukaryotes examined to date [59,79]. *D. pulex *has one SPO11 homolog which is present between the arthropod and vertebrate SPO11 clades in the phylogeny (Fig. 6A).

Following DSB formation, several meiosis-specific proteins act in concert to promote chromosomal synapsis and recombination. Genes encoding meiosis-specific proteins in our inventory are *DMC1 *(meiosis-specific paralog of *RAD51*), *MND1 *(also called *GAJ*) and *HOP2*; we also include *RAD54 *and its meiotic paralog *RAD54B *(Table 2). *RAD51 *and *DMC1 *are the two major eukaryotic homologs of eubacterial *recA *[80,81]. RAD51 is required for mitotic recombination, DNA damage repair and meiotic recombination, while DMC1 is meiosis-specific, required only for meiotic recombination and for normal synaptonemal complex (SC) formation [82,83]. RAD51 and DMC1 co-localize during meiosis and work together during meiotic recombination [84,85]. MND1 and HOP2 form a heterodimeric complex that interacts with RAD51 and DMC1 to promote interhomolog meiotic recombination and to reduce synapsis and recombination of non-homologous chromosomes [86,87]. RAD54B (also called RDH54 or TID1 in fungi) interacts with RAD54 during meiosis to stimulate D-loop formation by RAD51 [88,89] and helps to stabilize the DMC1-ssDNA complex in humans [90,91]. While these proteins are meiosis-specific, non-meiotic expression has been detected for *MND1*, *HOP2 *and *RAD54B*, but only in some mammals [90-93].

There are single copies of genes for MND1 and HOP2 in *D. pulex *(Additional File 2). The phylogenies (Fig. 6B, C) show *D. pulex *MND1 and HOP2 proteins are closely related to their respective homologs in arthropods. Single copies of genes for RAD54 and RAD54B are present in *D. pulex *(Fig. 6D). In the phylogeny, *D. pulex *RAD54 (306273) is present among animal orthologs but does not show a strong relationship to arthropods. *RAD54B *(207797) appears to have been lost in insects, so the presence of this gene in *D. pulex *suggests the loss might not be widespread in arthropods.

With genes encoding MND1, HOP2, RAD54, and RAD54B present in *D. pulex*, genes for RAD51 and DMC1 are expected to also be present due to the important interactions among these proteins in meiosis. While a single *RAD51 *homolog is present in *D. pulex*, meiosis-specific *DMC1 *appears to be absent (Fig. 6E). To address the possibility that the *Daphnia DMC1 *homolog was simply overlooked in the *D. pulex *genome database, we searched *D. pulex *ESTs at wFleabase [94] but did not find *DMC1 *transcripts. Attempts to amplify *DMC1 *from *D. pulex *using degenerate PCR with primers that amplify *DMC1 *(and *RAD51*) in a wide diversity of eukaryotes ([95]; Logsdon et al. unpublished) were also unsuccessful. Lastly, we searched for other eukaryotic RAD51-like family members (RAD51B, RAD51C, RAD51D, XRCC2 *and *XRCC3) that are more distantly related than DMC1 to RAD51 [96]. If we could identify these more distantly related and divergent protein sequences, then we should be able to find the gene for DMC1 if it is indeed present. Single copies of genes for XRCC2, RAD51C and RAD51D were found in *D. pulex *(Fig. 6E), although *RAD51B *(which, among animals, is only found in vertebrates) and *XRCC3 *appear to be absent. This strengthens the validity of the *DMC1 *absence in *D. pulex *and it is unlikely that a cryptic unsequenced or unassembled copy remains to be found.

The absence of *DMC1 *is scattered, but not widespread throughout arthropods; *DMC1 *homologs are absent in *Drosophila *and *Anopheles *[59], but present in the insects *Bombyx, Tribolium *and *Rhipicephalus *(Fig. 6E). Determining the ubiquity of the *DMC1 *loss within Branchiopods, Crustacea and Arthropods will shed light on the evolution of the machinery for homologous recombination in meiosis and possibly cyclical parthenogenesis. Among taxa with sequenced genomes, the only other case where *DMC1 *is absent while *RAD51, MND1 *and *HOP2 *are present is the microsporidian fungus *Encephalitozoon *[59]. However, little is known about microsporidian meiosis so the implications of the *DMC1 *loss are unclear. Similarly, meiosis and parthenogenesis in *D. pulex *are not fully understood so this result certainly merits further investigation.

##### C) Mismatch repair genes: MutS and MutL homologs

The eukaryotic homologs of bacterial MutS and MutL proteins form heterodimers that are involved in chromosomal synapsis, recombination and mismatch repair (MMR). In eukaryotes, there are up to seven *MutS *homologs (*MSH1-7*); we did not search for *MSH1 *(required for mitochondrial DNA stability in yeast) and *MSH7 *(specific to plants) in *D. pulex *(Table 2). MSH2 forms heterodimers with MSH3 and MSH6. MSH2/MSH6 (MutSα) tends to be the most abundant MutS heterodimer and is involved in the repair of short base-base mismatches and indels while MSH2/MSH3 (MutSβ) repairs longer mismatches (reviewed by [97]). The MSH4/MSH5 heteroduplex is meiosis-specific and has a unique function among eukaryotic *mutS *homologs in recognizing Holliday junctions and stabilizing heteroduplex formation during meiotic crossing-over and recombination [98]. MSH4 has also been shown to interact with RAD51 and DMC1 in mammalian meiosis [99].

The phylogeny of animal and fungal MutS homologs reveals five strongly-supported clades specific for each *MSH *gene (Fig. 7A). The topology is consistent with other phylogenies that group these five *MSH *genes together [100]. Our phylogeny supports the idea that separate duplications yielded the *MSH3/6 *and *MSH2/4/5 *lineages [101], although the exact branching order of the *MSH2*, *MSH4 *and *MSH5 *clades is not resolved. Single copies of genes for each MutS homolog are present in *D. pulex*, including the meiosis-specific *MSH4 *and *MSH5 *(Fig. 7A; Additional File 2). This shows the *MSH4 *and *MSH5 *loss in *Drosophila *is not widespread in arthropods since orthologs are present in *D. pulex *and in other insects (*Aedes, Anopheles *and *Apis*). The presence of *MSH3 *in *D. pulex *and the basal metazoan *Nematostella *indicates that *MSH3 *may have been lost in insects but retained in other arthropods and animals. Further taxon sampling in arthropods and other invertebrates is necessary to understand the extent of this gene loss. Although two additional partial "copies" of *MSH3 *on scaffold 1273 can be identified by BLAST, these most likely are mis-assemblies because they are truncated proteins flanked by repeats and they are 100% identical to protein 327819.

There are four eukaryotic homologs of bacterial *mutL *genes: *MLH1*, *MLH2*, *MLH3 *and *PMS1 *(here, we use the fungal designations for *MLH2 *and *PMS1*, which are confusingly referred to as *PMS1 *and *PMS2*, respectively, in animals; Table 2). MutL homologs form three heterodimers in which MLH1 is the common subunit [102]. MLH1/PMS1 (MutL-α) is the most abundant heterodimer in human mitotic cells [103] and functions in MMR [104], but also has a role in meiotic recombination [105]. The role of MLH1/MLH2 (MutL-β) in MMR is not well understood, while MLH1/MLH3 (MutL-γ) is involved in meiotic recombination [106,107] and MMR *in vitro *[108,109].

In the animal and fungal MLH phylogeny (Fig. 7B), four clades are resolved, one for each *MLH *gene. Three clades (*MLH1*, *PMS1 *and *MLH3*) are strongly supported but the overall branching order for *MLH1*, *MLH2, MLH3 *and *PMS1 *is unresolved. Weaker support for the *MLH2 *animal/fungal clade is likely due to rapidly evolving fungal sequences; when they are removed all four *MLH *clades are strongly supported (not shown). In *D. pulex*, there are single copies of each *MLH *homolog (Fig. 7B). The *MLH1 *copy (for protein 308451) is present within the MLH1 clade but does not show a strong relationship to other animals; however, neither do the other arthropod MLH1 sequences. *MLH2 *and *MLH3 *have been lost from the insects examined but are present in *D. pulex *and *Nematostella*, suggesting independent losses of these genes in insects. Thus, while insects are unable to form MutL-β or MutL-γ heterodimers, they can presumably still produce MutL-α, which is the most abundant and may have a minor role in meiotic recombination.

##### D) Expression patterns for meiosis-specific genes

Among the cohesin genes in *D. pulex*, EST and/or tiling array data provide evidence for expression of *SMC1 *and *SMC3 *copies (and the other non-cohesins *SMC *genes), for *RAD21 *and the three *REC8 *copies and for all five *SA *copies. Our RT-PCR results show the three *REC8 *copies are expressed in ovaries of both parthenogenetic cultures and in males, but not in female soma (Additional File 2). For the *SA *genes, we also found expression evidence for *SA-A, SA-B *and *SA-C *by RT-PCR in female gonads and soma and in males (Additional File 2) but no expression for *SA-D *or *SA-E*.

Among the interhomolog recombination genes, tiling array expression data validates the gene model for *SPO11 *in *D. pulex*. We also detected *SPO11 *transcription in males and in ovaries of both cyclical and obligate asexuals, but not in female soma (Additional File 2). There is also expression evidence for *MND1 *and *HOP2 *from EST and/or tiling arrays (Additional File 2). Our RT-PCR experiments also show *MND1 *and *HOP2 *expression in cyclical and obligate parthenogens, but *MND1 *was also expressed in males and in female soma. This result, together with non-meiotic expression of *MND1 *and *HOP2 *in some mammals [92,93], could suggest these genes are constitutively expressed in some species, either because they have an uncharacterized non-meiotic role or they are nonspecifically expressed at low levels. There is tiling array and/or EST expression data in *D. pulex *for all five *MSH *genes, for each *MLH *homolog, and for *RAD54 *and *RAD54B *(Additional File 2). RT-PCR for *MSH4 *and *MSH5 *show that these genes are expressed during cyclical and obligate parthenogenesis and also in males; only *MSH4 *expression was detected in female soma.

## Conclusion

### A Role for Meiotic Genes in Parthenogenesis?

The main objective of our meiotic gene inventory is to determine which genes necessary for meiosis are present in *D. pulex*. This information, along with gene expression patterns, can then be used to illuminate mechanistic differences between meiosis and both cyclical and obligate parthenogenesis in *D. pulex*. We emphasize that differences between parthenogenesis and meiosis are likely to relate to changes in: i) kinetochore orientation, ii) recombination bias and iii) sister chromatid cohesion [24,31]. From our gene inventory, the majority of meiotic genes are present in multiple copies in *D. pulex *(Table 2 and Additional File 2), which is also consistent with the high tandem gene content characteristic of the *D. pulex *genome. We speculate that these extra gene copies may be partly responsible for changes to these meiotic processes, as suggested by our model for parthenogenesis (Fig. 1). We suggest that some of these duplicated genes have the potential to serve parthenogenesis-specific functions, although it is possible that some duplicate gene copies have maintained their ancestral meiotic function. Below, we discuss implications that our meiotic gene inventory and expression data may have on understanding the mechanisms of parthenogenesis in *D. pulex*. We also incorporate these findings into a model highlighting the stages in meiosis where these genes could play important roles in the transition from meiosis to parthenogenesis.

According to our model (Fig. 1), stem cell division and maintenance are similar in meiosis and parthenogenesis. However, we invoke important changes in heterochromatin formation (especially at centromeres) and kinetochore attachment during parthenogenesis. PIWI subfamily proteins affect heterochromatin assembly and in *D. pulex *there are six PIWI subfamily gene copies (*AUB-A *to *AUB-F*), including two copies (*AUB-E *and *AUB-F*) expressed in ovaries but not in males or soma. Comparisons of expression patterns for these gene copies during meiosis and parthenogenesis may indicate potentially important roles for this protein family in both meiosis and parthenogenetic reproduction.

Polo kinases (PLKs) have diverse roles in meiosis and also in regulating the cell cycle, kinetochore formation and cohesin attachment and removal. *D. pulex *has at least three copies of *PLK1 *and several partial *PLK1*-like sequences. PLK1 is involved in orienting kinetochores during mitosis and meiosis. In *D. pulex *some PLK1 copies may be involved in altering microtubule attachment during parthenogenesis to allow amphitelic (like in mitosis and meiosis II) rather than syntelic (as in meiosis I) attachment of kinetochores (Fig. 1). Genes encoding some proteins that PLK1 interacts with are also present in multiple copies in *D. pulex*: PLK1 together with cyclin B (up to six gene copies in *D. pulex*) affects cell cycle regulation [31]. Since parthenogenesis in *D. pulex *is distinct from mitosis and meiosis, parthenogenetic cell cycle regulation may require interactions between specific cell cycle proteins (such as multiple gene copies for PLK1 and cyclin B) for successful cytokinesis.

According to our model, parthenogenetic sister chromatid cohesion must differ from cohesion during regular meiosis. The stepwise removal of cohesin in mitosis requires PLK1 to phosphorylate the SA subunit (which has five gene copies in *D. pulex*); changes in the timing of cohesin removal during parthenogenesis could be achieved by the activity of a "parthenogenetic" PLK1 copy that phosphorylates SA (perhaps also present as a parthenogenesis-specific copy) in a mitotic rather than meiotic fashion. Parthenogenetic-specific copies of TIMELESS/TIM-1 and TIMEOUT/TIM-2 may also be involved in the initial loading of cohesin during meiosis and parthenogenesis, as demonstrated in *C. elegans *for TIM-1 [48]. Distinct parthenogenetic cohesin complexes might also be present in *D. pulex*. If so, we would expect to find multiple copies of genes encoding cohesin complex proteins and those that are involved in its loading, targeting, and removal from chromosomes. In *D. pulex*, there are two gene copies each for SMC1 and SMC3 proteins which could represent the gain of a parthenogenesis-specific function in one copy (similar to the SMC1 and meiosis-specific SMC1β duplication in vertebrates [75]). There are also three copies of meiosis-specific *REC8 *and only one copy of its paralog *RAD21*. Some REC8 copies may be involved in differential regulation of cohesin removal (*e.g. *by separase cleavage) in parthenogenesis compared to meiosis. However, such divergent roles for REC8 are unknown; for example, three *REC8 *copies are also present in *C. elegans*, but their functional differences have not been extensively characterized [110].

The richness of cohesin complex genes in *D. pulex *is unique among eukaryotes. We speculate that the extra gene copies encode proteins that are involved in altering sister chromatid cohesion during parthenogenesis. Combinations of the various SMC1, SMC3, REC8 and SA copies could plausibly comprise different cohesin complexes with specific roles in meiosis and parthenogenesis. For example, our model for parthenogenesis posits meiosis-like cohesion during prophase (which allows homolog synapsis and SC formation) followed by a shift in favor of the function of the "parthenogenetic" cohesin (Fig. 1). At this point, centromeric cohesin would be released and sister chromatids, instead of homologs, would segregate, recapitulating the maternal genotype in the daughter cells in a mitosis-like division. This intriguing prospect will require detailed genetic screening to determine the functions of these genes. Alternatively, a parthenogenetic-specific cohesin might not replace RAD21 with REC8; during meiosis in *rec8*Δ yeast, RAD21 cohesin can establish amphitelic kinetochore attachment and loss of centromeric cohesin during anaphase I [111]. In essence, this resembles a mitotic division during meiosis I as suggested in our model for parthenogenesis. However, all three copies of *REC8 *are expressed during parthenogenesis (Table 1), so while some, or all, copies are likely involved in this process, RAD21 may also play an important role.

Our model also suggests that during parthenogenesis there is a change in recombination bias from between homologs to either between sister chromatids, or to no homologous recombination at all. This idea is supported by a study of mutation-accumulation lines in *D. pulex *showing that the loss of heterozygosity by ameiotic recombination was orders of magnitude greater than nucleotide mutation rates [16]; this clearly indicates that some degree of non-reciprocal recombination normally occurs during parthenogenetic reproduction. In our survey, the majority of genes involved in DSB formation, recombination initiation, promoting strand invasion and resolving Holliday junctions (*SPO11, MND1, HOP2, RAD54, RAD54B *and *RAD51*, along with *MSH *and *MLH *homologs) are present as single copies. This pattern implies that a parthenogenesis-specific function for these proteins is unlikely, or alternatively that the proteins do not function during parthenogenesis at all. The lack of variation in copy number of genes involved in HR and MMR may also reflect the importance of these pathways for the survival of *D. pulex*. Conversely, there are seven gene copies homologous to *RECQ2 *(*BLM*) which encodes a protein that limits crossing over and promotes Holliday junction resolution without reciprocal exchange. This contrast is striking: genes encoding proteins that promote meiotic recombination are present as single copies while a protein that suppresses crossing-over has seven gene copies. This may help to explain how meiotic recombination could be suppressed or altered during parthenogenesis. For example, if the single-copy genes maintain their function in meiosis (and mitosis, where applicable) while others such as RECQ2 copies have evolved novel roles unique to parthenogenesis, the result could be decreased levels or the absence of reciprocal recombination during parthenogenesis as predicted in our model.

The absence of meiosis-specific *DMC1 *could also have implications regarding innovations for recombination in meiosis and parthenogenesis in *D. pulex*. Increased frequency of interhomolog over intersister recombination is a defining characteristic of meiosis, a bias which is strongly enforced by DMC1 [80]. Thus, *D. pulex *has the machinery for synapsis but is missing the recombinase (and associated homolog bias) typically associated with this system. However, other meiotically competent animals missing *DMC1*, in addition to *MND1 *and *HOP2 *(*e.g. D. melanogaster *and *C. elegans*), have presumably evolved unique innovations (not yet understood) to compensate for the absence of these genes. Another possibility for *D. pulex *is the promotion of homolog bias during meiosis by the SC. Although well-conserved structurally, the rapid evolution of SC proteins (such as HOP1, which we were unable to find in *D. pulex*) make them difficult to identify. Further study of synapsis and recombination control in *Daphnia *provides an interesting comparative system to better understand these processes in animals.

Our RT-PCR with *D. pulex *cultures detected expression for 44 meiotic gene copies during oogenesis in cyclical parthenogenetic cultures and also during both sexual and obligate asexual reproduction and in female soma (Table 1, Additional File 2). Initially, we found that presence and absence of 25 meiotic genes during meiosis (resting egg production) and parthenogenesis (direct-developing egg production) in a cyclically parthenogenetic culture were indistinguishable. That is, gene expression specific to either meiosis or direct developing egg production (*i.e. *ovaries from cyclically parthenogenetic cultures) was not observed for these genes (Table 1). In addition, our RT-PCR experiments in cyclical and obligate parthenogenetic cultures for the >40 genes that we subjected to more detailed phylogenetic analyses (Additional File 2) did not detect expression specific to sexual tissues (meiotic ovaries) or obligate asexual tissues (ovaries from obligate parthenogenetic cultures). Several genes were found to have expression restricted to the germline or soma.

However, transcript levels may not be an accurate measure of protein function during parthenogenesis, and although meiosis genes are expressed during parthenogenesis, it is possible that their function is altered or absent. Expression patterns were also not determined for all gene copies in this study, so perhaps some of these may exhibit parthenogenesis-specific expression patterns when examined more closely. In addition, mature and immature oocyte clusters can exist within the same ovary, yet be undergoing two different reproductive modes (*e.g. *meiosis and parthenogenesis; [15]). Thus, a technique with increased spatial resolution, such as *in situ *hybridization, may be required to detect qualitatively important differences in expression. Yet, many gene copies were expressed in ovaries undergoing either obligate parthenogenesis or meiosis, indicating that these genes could function during both processes. There were also several genes expressed in female soma. Interestingly, expression of *MND1 *and *MSH4 *in female soma could indicate that these genes are not meiosis-specific and may have uncharacterized non-meiotic (*i.e. *non-reproductive) role(s). This could also represent low level constitutive expression, as non-meiotic gene expression has been detected for *MND1 *(and *HOP2*) in mammals [92,93]. Among genes not expressed, *POLO-J, POLO-K, RECQ2-B, TIM-F *and *TIM-G *are likely pseudogenes based on both lack of expression and other characteristics in gene architecture (*e.g. *stop codons). EST and/or tiling array expression evidence for *RECQ2-B, RECQ2-C, POLO-F, SA-D *and *SA-E *contradicts the absence of expression from our RT-PCR results, although the causes of these discrepancies are unclear.

Comparative data from other *Daphnia *species and parthenogenetic taxa will help to clarify the deeper history of reproductive gene duplications in this genus. *Apis mellifera *(honeybee) is the only other animal in our phylogenetic analyses that regularly undergoes parthenogenesis and that also has an assembled genome. The genome of the pea aphid *Acyrthosiphon pisum *is currently being sequenced, and it will be most interesting to compare the meiotic gene inventory of this cyclical parthenogen to that of *D. pulex*. Reproduction in *Apis *(as in many hymenopterans) is arrhenotokous, meaning haploid males are parthenogenetically produced while fertilized eggs turn into diploid females. This differs from cyclical parthenogenesis (or heterogony) in *Daphnia *which alternates between parthenogenesis (where both females and males are produced by parthenogenesis) and sexual reproduction. We did not find any striking expansions in gene copy number or conspicuous gene absences in our survey of meiotic genes in *Apis*. Thus, while both taxa have parthenogenetic phases of their life cycles, the meiotic gene catalog in *D. pulex *is markedly enriched for gene duplications compared to *Apis*. Whether this is mechanistically or causally related to differences between cyclical parthenogenesis and arrhenotoky is unclear.

Our meiotic gene inventory has identified expansions in particular meiotic genes and gene families that we speculate are related to the mechanism of parthenogenesis in *D. pulex*. From our analysis, we cannot make substantial conclusions on parthenogenetic-specific functions for these gene copies without extensive expression and functional assays in D. pulex. However, considering the multi-functional roles of many of the proteins in our inventory (e.g. PLK-1), the acquisition of an additional yet-to-be characterized parthenogenetic-specific function would not be unreasonable to consider. It is clear that tandem duplications are widespread in the *D. pulex *genome and to a much greater extent than other sequenced invertebrates. However, at this point we cannot distinguish whether tandem duplications are the consequence or cause of parthenogenesis. For example, if cyclical parthenogenesis has a higher rate of unequal crossing over or slip-strand mispairing than does meiosis, the consequence could be a higher frequency of tandem duplications during parthenogenesis; thus, a large proportion of duplicate genes would be expected to have arisen since the origin of parthenogenesis in this species. Alternatively, duplicated meiotic genes might be the cause of parthenogenesis by driving the genetic sub-functionalization of different biochemical activities associated with parthenogenetic production of either direct-developing (cyclical) or diapause (obligate) oocytes.

One approach to resolving the issue of cause or effect would be to date the duplications by comparing the relative ages of tandem gene pairs in *Daphnia *associated with meiosis to the ages of tandem duplicate genes not associated with meiosis. If gene families associated with meiosis have a significantly older distribution than gene families that are not, this would indicate that meiotic gene duplications preceded the origin of parthenogenesis, while a younger age would suggest that duplications of meiotic genes have been a consequence of parthenogenesis. One way to accomplish this is to complete a phylogenetic survey of duplicated meiotic genes throughout the Cladocera, all of which reproduce *via *cyclical parthenogenesis, and in the clam shrimp *Cyclestheria *which may represent the closest extant representative of the lineage from which cladocerans arose [112]. If multiple meiotic gene copies are also present throughout Cladocera and in *Cyclestheria *then this would suggest meiotic gene duplications might have coincided with the origin of cyclical parthenogenesis and indicate the minimum complement of duplicated meiotic genes that are required for cyclical parthenogenesis. A sporadic distribution of duplicated genes could suggest ancestral duplications and multiple losses or independent origins of duplicated gene. Such studies require an in-depth analysis of the age distribution of gene duplications, tandem and otherwise, which is outside the scope of this report.

## Methods

### Phylogenetic Analysis

To find homologs for each gene of interest in *Daphnia*, protein sequences from *Drosophila *and other metazoans were used as queries in BLASTP and TBLASTN searches against the *D. pulex *genome at JGI [113] and wFleabase [94] and putative protein homologs were retrieved. For each gene, amino acid alignments that included putative homologs from *D. pulex *and from a diverse sampling of metazoans (and, in some cases, fungi and plants) were constructed using Clustal X [114] and edited manually using MACCLADE 4.08 [115]. Phylogenetic analyses were done using MrBayes 3.1.2 [116] with the WAG + I + 8G substitution model [117]. Four Markov chains were used (one heated and three cold) and the analysis was run for one million generations with trees sampled every 1000 generations. From a plot of likelihood scores versus generation, the burnin value was determined for each analysis and only trees from that point on with the best posterior probabilities were retained for constructing the consensus tree which was edited with TREETOOL.

### Gene Expression analysis

*Daphnia *cultures were collected between 2001–2004 and genotyped using allozymes and mtDNA (see [19] for details). For gene expression studies, *D. pulex *were reared in filtered pond water at 18°C on a 12:12 cycle of light:dark and fed *Scenedesmus *algae (0.5 mg/ml) every two days. Prior to sacrifice, animals were inspected by microscopy to verify sex and reproductive status of females. Females were scored as obligate asexuals according to whether they could produce viable resting eggs in the absence of males. Whole males were used, and fully vitellogenic ovaries were dissected from 10 cyclical and 10 obligate parthenogenetic females making resting eggs by fixing and dissecting in 80% ethanol. Somatic tissues from the same animals were segregated from the gonads, and total RNA was isolated by removing the ethanol, incubating in 100 μl of lysis buffer (PicoPure kit; Arcturus, Inc.) at 42°C for 30 min, and adding 100 μl of 70% ethanol. This mixture was then column purified according to the manufacturer's protocol, including a DNase digestion step.

Prior to reverse transcription, RNA samples were assessed by capillary electrophoresis using a Bioanalyzer 2100 (Agilent), showing intact ribosomal RNA bands that indicate minimal RNA degradation in each case. Total RNA (less than 1 μg each) was added to 100 ng oligo-dT and 0.4 mM dNTPs, incubated at 65°C for 5 min and quick chilled. The reactions were added to 1× (final concentration) first strand buffer (Invitrogen), 10 mM DTT, 1 μl of RNasin (Ambion) and 5 units of SuperScript II (Invitrogen), and incubated at 48°C for 60 min and 70°C for 15 min. Reactions were brought to 50 μl total with 10 mM Tris, pH 8.0. Negative controls were performed by adding water instead of reverse transcriptase, and failed to amplify control primers in all cases, indicating undetectable genomic DNA contamination.

Polymerase chain reaction (PCR) amplification was conducted using Taq polymerase (BioLine) or Vent polymerase (New England Biolabs) with cycling parameters specific for each primer pair tested (see Supplementary Table 1 for primer sequences and theoretical melting temperatures). Products were visualized on 1.4% TBE agarose gels stained with 10 μg/ml ethidium bromide. For cloning of PCR products, Vent-amplified reactions were incubated with exo-Taq and 1 mM dATP prior to incubation with Topo pCR-II TA-vector (Invitrogen). Cloning was conducted according to manufacturer's instructions using chemically competent DH5a cells. Plasmid DNA was recovered from transformed colonies using PureLink miniprep kits (Invitrogen). Sequencing of PCR products (200 ng) or purified plasmid (50 ng) was done with BigDye v.3 (Applied Biosystems) on an ABI 3730 sequencer at the Indiana Molecular Biology Institute (Indiana University).

## Abbreviations

HR: Homologous Recombination; GSC: Germline Stem Cell; BLAST: Basic Local Alignment Search Tool; EST: Expressed Sequence Tag; RT-PCR: Reverse Transcription Polymerase Chain Reaction; MMR: Mismatch Repair; SC: Synaptonemal Complex; CO: Crossover; NCO: Non-crossover; SDSA: Synthesis Dependent Strand Annealing; aa: amino acids; DSB: Double Strand Break; ORF: Open Reading Frame; dHJ: double Holliday Junction.

## Authors' contributions

AS, JL and BE designed the gene inventory, interpreted results and drafted the manuscript. AS and BE performed BLAST searches and manual gene annotations. AS implemented phylogenetic analyses. BE conceived the study and performed RT-PCR experiments. All authors read and approved the final manuscript.

## Supplementary Material

Additional file 1 **Representative images of RT-PCR products (from Additional File **2**) subjected to gel electrophoresis**. Contains gel images of RT-PCR products amplified with primers detailed in Additional File 3. Click here for file

Additional file 2 **Annotated genes in *Daphnia pulex *associated with meiosis**. Protein IDs correspond to gene models assigned by the Joint Genome Institute [113] and their corresponding genomic coordinates. Columns for Meiotic Ovaries (resting egg production by cyclical parthenogens), Obligate Parthenogenetic Ovaries (resting egg production by obligate parthenogens), Males (meiosis; whole animals) and Female Soma (cyclical parthenogens) represent RT-PCR data from these tissues as presence (+) or absence (-) using primers detailed in Additional File 2. EST, Tiling path, and repeat region data for each protein were found on the wFleabase genome browser [94]. N/D is not determined. Click here for file

Additional file 3 **Primers used in RT-PCR gene expression studies**. Primer sequences, protein ID and genomic coordinates for RT-PCR target genes. Click here for file

Additional file 4 **Gene expression patterns and genomic locations corresponding to truncated copies of SMC1 proteins in *D. pulex***. Contains information about SMC1 psuedogenes and truncated proteins, including RT-PCR and tiling path microarray gene expression data. Click here for file

Additional file 5 **Genomic locations of two repetitive regions found near the *D. pulex *Rec8 paralogs A, B and C**. Contains coordinates of repetitive regions near paralogs of meiotic cohesin Rec8. Click here for file
