# Supplementary material for: Meiosis genes in Daphnia pulex and the role of parthenogenesis in genome evolution
Source: BMC Evol Biol. 2009 Apr 21;9:78. doi: 10.1186/1471-2148-9-78 (PMC2680839; doi:10.1186/1471-2148-9-78)
Supplement: Additional file 1 — Representative images of RT-PCR products (from Additional File 2) subjected to gel electrophoresis. Contains gel images of RT-PCR products amplified with primers detailed in Additional File 3. [file 1471-2148-9-78-S1.doc]

Additional File 1. Representative examples of RT-PCR products visualized on agarose gels. Note that replicate reactions were performed, only one of which is shown for each sample here. In the case of discrepant samples, additional biological replicates were performed. In this notation, A is cDNA template obtained from ovaries of obligate asexual females producing resting eggs parthenogenetically, S is cDNA template obtained from ovaries of cyclical parthenogen (sexual) females producing resting eggs via meiosis, and M is whole males. Primers are detailed in Supplemental Table 1. Top panel lanes: M=100 bp marker; 1=S AUB-B 2=A AUB-B 3=S AUB-D 4=A AUB-D 5=S SPO11 6=A SPO11 7=S HOP2 8=A HOP2 9=S MSH4 10=A MSH4 11=S MSH5 12=A MSH5 13=S SA-A 14=A SA-A 15=S SA-A (replicate 2) 16=A SA-B 17=S SA-B. Bottom Panel lanes: 1=M RECQ4 2=S RECQ4 3=A RECQ4 4=M RECQ5 5=S RECQ5 6=A RECQ5 7=M POLO-A 8=S POLO-A 9=A POLO-A 10=M POLO-B 11=S POLO-B 12=A POLO=B 13=AUB-D 14=S AUB-D 15=AUB-D 16=M AUB-E 17=S AUB-E 18=A AUB-E 19=M AUB-F 20=S AUB-F 21=A AUB-F 22=M AGO3 23=S AGO3 24=A AGO3 25=M TIM-C 26=S TIM-C 27=A TIM-C 28=M TIM-C 29=S TIM-C 30=A TIM-C. (Lane 30 shows a failed reaction that when replicated had a band present at 300 bp).
