# Supplementary material for: Meiosis genes in Daphnia pulex and the role of parthenogenesis in genome evolution
Source: BMC Evol Biol. 2009 Apr 21;9:78. doi: 10.1186/1471-2148-9-78 (PMC2680839; doi:10.1186/1471-2148-9-78)
Supplement: Additional file 2 — Annotated genes in Daphnia pulex associated with meiosis. Protein IDs correspond to gene models assigned by the Joint Genome Institute [113] and their corresponding genomic coordinates. Columns for Meiotic Ovaries (resting egg production by cyclical parthenogens), Obligate Parthenogenetic Ovaries (resting egg production by obligate parthenogens), Males (meiosis; whole animals) and Female Soma (cyclical parthenogens) represent RT-PCR data from these tissues as presence (+) or absence (-) using primers detailed in Additional File 2. EST, Tiling path, and repeat region data for each protein were found on the wFleabase genome browser [94]. N/D is not determined. [file 1471-2148-9-78-S2.doc]

Additional File 2. Annotated genes in *Daphnia pulex* associated with meiosis.

| **Gene Name** | **Protein ID** | **Scaffold Coordinates** | **Meiotic Ovaries** | **Obligate Parthen-ogenetic Ovaries** | **Males** | **Female Soma** | **EST** | **Tiling Path** |
| --- | --- | --- | --- | --- | --- | --- | --- | --- |
| *AUB-A* | 239845 | 14:1485404-1488954 | N/D | N/D | N/D | N/D | + | + |
| *AUB-B* | 220987 | 3:3120683-3124261 | + | + | + | + | + | + |
| *AUB-C* | 308681 | 83:137847-141473 | + | + | + | + | + | + |
| *AUB-D* | 240344 | 16: 324312-328229 | + | + | + | − | − | + |
| *AUB-E* | 100816 | 16: 318792-323251 | + | + | − | − | + | + |
| *AUB-F* | 195225 | 16: 328899-332239 | + | + | − | − | + | + |
| *AGO3* | 442510 | 20:1003921-1007807 | + | + | + | + | + | + |
| *AGO1* | 305022 | 32:50955-56189 | N/D | N/D | N/D | N/D | + | + |
| *AGO2* | 311791 | 4:2913591-2917411 | N/D | N/D | N/D | N/D | + | + |
| *AGO* | 442513 | 102:244960-251030 | N/D | N/D | N/D | N/D | + | + |
| *AGO* | 130069 | 120:17503-18656 | N/D | N/D | N/D | N/D | − | − |
| *AGO* | 317739 | 22:94055-94838 | N/D | N/D | N/D | N/D | + | + |
| *Cyclin A* | 216737 | 88: 274712-277003 | N/D | N/D | N/D | N/D | + | + |
| *Cyclin B* copy A | 206150 | 3:445384-447543 | N/D | N/D | N/D | N/D | + | + |
| *Cyclin B* copy B | 299529 | 100:431012-432846 | N/D | N/D | N/D | N/D | + | + |
| *Cyclin B* copy C | 442518 | 49: 485562-486686 | N/D | N/D | N/D | N/D | + | − |
| *Cyclin B* copy D | 222925 | 12:754695-756976 | N/D | N/D | N/D | N/D | + | + |
| *Cyclin B* copy E | 299508 | 35: 456503-457980 | N/D | N/D | N/D | N/D | − | − |
| *Cyclin B3* | 210441 | 17: 647208-649684 | N/D | N/D | N/D | N/D | + | + |
| *Cyclin D*, copy A | 299604 | 1:410344-412996 | N/D | N/D | N/D | N/D | + | + |
| *Cyclin D*, copy B | 46610 | 11: 1856476-1861733 | N/D | N/D | N/D | N/D | − | + |
| *Cyclin E* | 299520 | 163:128426-125621 | N/D | N/D | N/D | N/D | + | + |
| *CDK1/CDC2* | 299546 | 79:381760-383475 | N/D | N/D | N/D | N/D | + | + |
| *CDK2* | 299469 | 77:77469-79151 | + | + | N/D | N/D | + | + |
| *CDK4* | 318234 | 24:373853-387839 | N/D | N/D | N/D | N/D | + | + |
| *CDK10* | 230543 | 10:925176-927941 | N/D | N/D | N/D | N/D | + | + |
| *POLO-A* | 309099 | 9:1434540-1437384 | + | + | + | + | + | + |
| *POLO-B* | 301125 | 12:1037337-1040137 | + | + | + | − | + | + |
| *POLO-C* | 237171 | 9:484138-487481 | − | − | − | − | + | + |
| *POLO-D* | 302355 | 17: 769975-773749 | + | + | + | − | + | + |
| *POLO-E* | 309383 | 97: 256695-259968 | + | + | + | − | + | + |
| *POLO-F* | 251822 | 61: 8853-11468 | − | − | − | − | − | + |
| *POLO-G* | 11646 | 382:26972-30731 | + | + | + | − | + | − |
| *POLO-H* | 334665 | 382:24234-25958 | N/D | N/D | N/D | N/D | + | + |
| *POLO-I* | 5682 | 382:24594-28798 | N/D | N/D | N/D | N/D | − | − |
| *POLO-J* | 262199 | 165: 170587-171600 | − | − | − | − | − | − |
| *POLO-K* | 323070 | 51: 759385-760291 | − | − | − | − | − | − |
| *MCM2* | 306072 | 44:872605-876279 | N/D | N/D | N/D | N/D | + | + |
| *MCM3* | 305021 | 32: 45513-49518 | N/D | N/D | N/D | N/D | + | + |
| *MCM4* | 329431 | 120:151712-155138 | N/D | N/D | N/D | N/D | + | + |
| *MCM5* | 227386 | 75:447869-451282 | N/D | N/D | N/D | N/D | + | + |
| *MCM6* | 307300 | 61:466975-470629 | N/D | N/D | N/D | N/D | + | + |
| *MCM7* | 301022 | 118:33191-36300 | N/D | N/D | N/D | N/D | + | + |
| *MCM8* | 62337 | 116:277106-280527 | N/D | N/D | N/D | N/D | + | − |
| *MCM9* | 322108 | 45: 126071-129633 | N/D | N/D | N/D | N/D | + | + |
| *TIM-A* | 382 | 25:47263-53103 | N/D | N/D | N/D | N/D | − | + |
| *TIM-B* | 265583 | 259:64799-70140 | N/D | N/D | N/D | N/D | − | − |
| *TIM-C* | 318263 | 24:606442-616374 | + | + | + | + | + | + |
| *TIM-D* | 103010 | 24:175930-179840 | + | + | − | − | − | − |
| *TIM-E* | 243157 | 24:181280-184538 | + | + | + | − | − | − |
| *TIM-F* | 103009 | 24:170734-171597 | − | − | − | − | − | − |
| *TIM-G* | 243162 | 24:201191-202787 | − | − | − | − | − | − |
| *TIM-H* | 309208 | 91:61662-67809 | N/D | N/D | N/D | N/D | − | − |
| *TIM-I* | 313767 | 10:18912-22562 | N/D | N/D | N/D | N/D | + | − |
| *TIM-J* | 308128 | 75:69226-71817 | N/D | N/D | N/D | N/D | + | + |
| *TIM-K* | 235857 | 6:1256392-1261444 | N/D | N/D | N/D | N/D | − | + |
| *TIM-L* | 307154 | 6:2000165-2005603 | N/D | N/D | N/D | N/D | + | − |
| *TIM-M* | 236054 | 6:2092018-2099144 | N/D | N/D | N/D | N/D | − | − |
| *RECQ1* | 313139 | 8:571640-596639 | + | + | + | + | + | + |
| *RECQ2-A* | 40427 | 2:2784790-2786929 | + | + | + | + | − | + |
| *RECQ2-B* | 47908 | 14: 1047836-1052516 | − | − | − | − | + | − |
| *RECQ2-C* | 246302 | 35:1004050-1007117 | − | − | − | − | + | − |
| *RECQ2-D* | 61614 | 102: 254297-257298 | + | + | − | − | − | − |
| *RECQ2-E* | 329283 | 117: 290762-292929 | + | + | − | − | − | − |
| *RECQ2-F* | 258939 | 118: 151216-155911 | − | − | − | − | − | − |
| *RECQ2-G* | 13555 | 387: 884-3709 | − | − | − | − | − | − |
| *RECQ4* | 347585 | 9: 780905-784938 | + | + | + | + | − | + |
| *RECQ5* | 347586 | 4:997088-1006264 | + | + | + | + | + | + |
| *SMC1A* | 318525 | 25:541672-546812 | N/D | N/D | N/D | N/D | + | + |
| *SMC1B* | 211085 | 20:564649-569968 | N/D | N/D | N/D | N/D | + | + |
| *SMC2* | 187038 | 2:1105636-1109827 | N/D | N/D | N/D | N/D | + | + |
| *SMC3A* | 317147 | 19:1150479-1156115 | N/D | N/D | N/D | N/D | + | + |
| *SMC3B* | 63318 | 138:30197-34838 | N/D | N/D | N/D | N/D | + | + |
| *SMC4* | 331955 | 177:193964-199013 | N/D | N/D | N/D | N/D | + | + |
| *SMC5* | 226104 | 47:649458-658022 | N/D | N/D | N/D | N/D | + | + |
| *SMC6A* | 103378 | 25:650825-655262 | N/D | N/D | N/D | N/D | + | + |
| *SMC6B* | 224715 | 25:788039-795256 | N/D | N/D | N/D | N/D | + | + |
| *RAD21* | 114887 | 141:52584-55706 | N/D | N/D | N/D | N/D | + | + |
| *REC8-A* | 221983 | 7:1508565-1514131 | + | + | + | − | + | + |
| *REC8-B* | 308284 | 77:576658-579947 | + | + | + | − | + | − |
| *REC8-C* | 308285 | 77:597511-594178 | + | + | + | − | + | − |
| *SA-A* | 347501 | 3:3693056-3696950 | + | + | + | + | + | + |
| *SA-B* | 221442 | 5:253009-258106 | + | + | + | + | + | + |
| *SA-C* | 95269 | 3:192949-196823 | + | + | + | + | + | + |
| *SA-D* | 95243 | 3:23625-27772 | − | − | − | − | + | + |
| *SA-E* | 41238 | 3:40593-43810 | − | − | − | − | + | + |
| *SPO11* | 299643 | 3:494509-496672 | + | + | + | − | − | + |
| *MND1* | 308088 | 74:422292-423779 | + | + | + | + | + | + |
| *HOP2* | 190785 | 2:2808348-2809289 | + | + | − | − | + | − |
| *RAD54* | 306273 | 47:911625-915094 | N/D | N/D | N/D | N/D | + | + |
| *RAD54B* | 207797 | 6:1648449-1652811 | N/D | N/D | N/D | N/D | − | + |
| *RAD51* | 299714 | 17:682544-680569 | N/D | N/D | N/D | N/D | + | + |
| *XRCC2* | 5268 | 163:214535-215111 | N/D | N/D | N/D | N/D | + | − |
| *RAD51C* | 242313 | 21:642866-644579 | N/D | N/D | N/D | N/D | + | + |
| *RAD51D* | 300755 | 11:1310009-1310993 | N/D | N/D | N/D | N/D | + | − |
| *MSH2* | 325677 | 72:191411-195574 | N/D | N/D | N/D | N/D | + | + |
| *MSH3* | 327819 | 95:356706-360856 | N/D | N/D | N/D | N/D | + | − |
| *MSH4* | 303339 | 20:685585-700925 | + | + | + | + | + | + |
| *MSH5* | 337363 | 1499:4612-8525 | + | + | + | − | − | + |
| *MSH6* | 303932 | 24:982188-986398 | N/D | N/D | N/D | N/D | + | + |
| *MLH1* | 308451 | 8:716995-719840 | N/D | N/D | N/D | N/D | + | + |
| *MLH2* | 326138 | 76:524487-528310 | N/D | N/D | N/D | N/D | + | − |
| *MLH3* | 324171 | 60:208317-211547 | N/D | N/D | N/D | N/D | + | + |
| *PMS1* | 311334 | 4:606526-609758 | N/D | N/D | N/D | N/D | + | + |

Protein IDs correspond to gene models assigned by the Joint Genome Institute [113] and their corresponding genomic coordinates. Columns for Meiotic Ovaries (resting egg production by cyclical parthenogens), Obligate Parthenogenetic Ovaries (resting egg production by obligate parthenogens), Males (meiosis; whole animals) and Female Soma (cyclical parthenogens) represent RT-PCR data from these tissues as presence (+) or absence (-) using primers detailed in Supplemental Table 1. EST, Tiling path, and repeat region data for each protein were found on the wFleabase genome browser [94]. N/D is not determined.
